# Molecular spectrum of *BRAF, NRAS* and *KRAS* gene mutations in plasma cell dyscrasias: implication for MEK-ERK pathway activation

**DOI:** 10.18632/oncotarget.4434

**Published:** 2015-06-10

**Authors:** Marta Lionetti, Marzia Barbieri, Katia Todoerti, Luca Agnelli, Simona Marzorati, Sonia Fabris, Gabriella Ciceri, Serena Galletti, Giulia Milesi, Martina Manzoni, Mara Mazzoni, Angela Greco, Giovanni Tonon, Pellegrino Musto, Luca Baldini, Antonino Neri

**Affiliations:** ^1^ Department of Clinical Sciences and Community Health, University of Milan, Milan, Italy; ^2^ Hematology Unit, Fondazione IRCCS Ca’ Granda, Ospedale Maggiore Policlinico, Milan, Italy; ^3^ Laboratory of Pre-Clinical and Translational Research, IRCCS-CROB, Referral Cancer Center of Basilicata, Rionero in Vulture, Potenza, Italy; ^4^ Molecular Mechanism Unit, Department of Experimental Oncology and Molecular Medicine, Fondazione IRCCS Istituto Nazionale dei Tumori, Milan, Italy; ^5^ Functional Genomics of Cancer Unit, Division of Experimental Oncology, San Raffaele Scientific Institute, Milan, Italy

**Keywords:** multiple myeloma, plasma cell leukemia, RAS, BRAF, next generation sequencing

## Abstract

Multiple myeloma (MM) is a clinically and genetically heterogeneous plasma cell (PC) malignancy. Whole-exome sequencing has identified therapeutically targetable mutations such as those in the mitogen-activated protein kinase (MAPK) pathway, which are the most prevalent MM mutations. We used deep sequencing to screen 167 representative patients with PC dyscrasias [132 with MM, 24 with primary PC leukemia (pPCL) and 11 with secondary PC leukemia (sPCL)] for mutations in *BRAF, NRAS* and *KRAS*, which were respectively found in 12%, 23.9% and 29.3% of cases. Overall, the MAPK pathway was affected in 57.5% of the patients (63.6% of those with sPCL, 59.8% of those with MM, and 41.7% of those with pPCL). The majority of *BRAF* variants were comparably expressed at transcript level. Additionally, gene expression profiling indicated the MAPK pathway is activated in mutated patients. Finally, we found that vemurafenib inhibition of BRAF activation in mutated U266 cells affected the expression of genes known to be associated with MM. Our data confirm and extend previous published evidence that MAPK pathway activation is recurrent in myeloma; the finding that it is mediated by *BRAF* mutations in a significant fraction of patients has potentially immediate clinical implications.

## INTRODUCTION

Multiple myeloma (MM) is a malignant disorder characterized by the clonal proliferation of bone marrow (BM) plasma cells (PCs). The genetic background and clinical course of the disease are highly heterogeneous, ranging from pre-malignant monoclonal gammopathy of undetermined significance to smoldering MM, symptomatic MM, and extra-medullary MM/plasma cell leukemia (PCL) [1]. Clinically, PCL has two forms: primary PCL (pPCL) originating *de novo*, or secondary PCL (sPCL) arising from a pre-existing MM [2]. Recent therapeutic advances have extended overall patient survival, but current anti-MM treatments are not specific and act by means of pleiotropic mechanisms. However, genome-wide next-generation sequencing (NGS) studies have provided a rationale for molecularly aimed treatment approaches by identifying specifically targetable mutations such as those in the mitogen-activated protein kinase (MAPK) pathway, which are the most prevalent mutations in MM [3-6]. Along with already known *NRAS* and *KRAS* mutations, these also include *BRAF* mutations, which have been recently reported to occur in 4-15% of patients [4-6] and may be of potentially immediate clinical relevance because of the availability of effective BRAF inhibitors that are also being investigated in MM treatment [7-9].

In this study, we used targeted NGS to screen a large and representative series of patients with intra- and extra-medullary MM (including pPCL or sPCL) for mutations in *BRAF, NRAS* and *KRAS*. We evaluated the relationships between the identified variants and the clinical and biological features of the disease, and determined the transcriptional signature associated with MAPK pathway activation in MM.

## RESULTS

### *BRAF* mutations in PC dyscrasias

In order to estimate the frequency of *BRAF* mutations in different forms of PC dyscrasias, 167 specimens (132 MM, 24 pPCLs and 11 sPCLs) and 21 HMCLs underwent NGS of the mutational hot-spots, namely exons 11 and 15 (Cosmic Release v70, at http://cancer.sanger.ac.uk/cancergenome/projects/cosmic/). The median depth of coverage was 233x (range 100-962x) and, after the exclusion of intronic, synonymous and germline variants, nine distinct single nucleotide variations (SNVs) were detected in 20 patients and one HMCL. All of the mutations were missense substitutions (Table 1) and their occurrence was confirmed in an independent PCR product in all cases. Variant allele frequencies (VAFs) ranged from 0.86% to 70.7% of total reads per sample (Supplementary Figure 1).

**Table 1:** Summary of non-synonymous *BRAF* variants identified by NGS

Variant (GRCh38)	AA change	dbSNP ID	COSMIC ID (v71)	MM literature	Mutated patients (percentage of mutated sequencing reads)						
140753336A>T	V600E	rs113488022	COSM476	ref 4, 5, 6, 24	MM-313 (44.59%)	PCL-043 (44.59%)	PCL-015 (41.18%)	MM-441 (35.74%)	MM-268 (29.57%)	MM-177 (27.18%)	MM-446 (26.48%)
140753355C>T	D594N	rs397516896	COSM27639	*	MM-295 (50.00%)	PCL-026 (43.05%)	MM-036 (5.61%)	MM-140 (1.68%)			
140781602C>G	G469A	rs121913355	COSM460	ref 5	PCL-023 (70.70%)	PCL-042 (51.26%)	MM-335 (50.52%)				
140753354T>C	D594G	rs121913338	COSM467	ref 4, 6	MM-435 (22.22%)	MM-219 (7.78%)	MM-411 (0.86%)				
140753332T>A	K601N	/	COSM6265	ref 5	U266 (65.62%)						
140753379C>T	E586K	rs121913340	COSM463	ref 4	PCL-026 (42.15%)						
140753349C>G	G596R	rs121913361	COSM469	/	MM-224 (35.44%)						
140753333T>G	K601T	rs397507484	COSM3878760	/	MM-039 (28.53%)						
140753353A>C	D594E	/	COSM253330	/	PCL-028 (4.65%)						

* Boyd EM *et al*. High resolution melting analysis for detection of BRAF exon 15 mutations in hairy cell leukaemia and other lymphoid malignancies. Br J Haematol. 2011; 155: 609-612.

Mutations were detected in 10.6% of the patients with MM at onset (14/132), 20.8% of pPCLs (5/24) and 9.1% of sPCLs (1/11). The main molecular features of the 20 *BRAF*-mutated patients are shown in Supplementary Table 1A; there was no significant association with any molecular lesion (Supplementary Table 1B). Five of these patients (four with MM and one with pPCL) carried a mutation whose VAF was below the Sanger detection limit (i.e. about 10% under our experimental conditions). The only mutation among the HMCLs was found in the U266 cell line, which harbored the K601N substitution in 65% of the sequencing reads, as previously reported [4-6].

**Figure 1:**
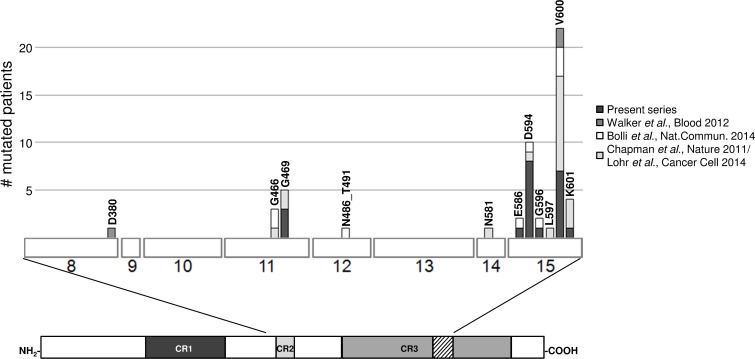
Compendium of the *BRAF* mutations in primary MM/PCL patients as found in the present series and other recent reports [4-6, 24] The three regions conserved in all RAF proteins (conserved region (CR) 1, CR2 and CR3) are shown beneath the main figure, with the activation segment within CR3 indicated by diagonal lines. Exons are numbered under the boxes.

The variants mainly targeted exon 15 (17/20, 85%) (Figure 1). The most frequent mutation was V600E (found in seven cases), which was reported to destabilize the hydrophobic interaction between the glycine-rich loop and the activation segment, thus locking the protein in its active conformation and increasing BRAF kinase activity [10, 11]. The same occurs in the case of the high activity mutant G469A [11] (found in three cases), whereas activation of the MEK-ERK signalling pathway by E586K (detected in one sample) could be mediated by the disruption of an intra-molecular regulatory interaction [12]. The G596R substitution (found in one case) impairs the kinase activity of BRAF, which cannot activate MEK directly but is still capable of activating MEK-ERK signalling by forming heterodimers with CRAF (which is activated in a RAS-independent manner), and also increases the activation of MEK-ERK signalling [12]. The K601 residue was targeted by two different mutations, K601N (in U266 cells) and K601T (in MM-039); to the best of our knowledge, no functional characterization of these mutants is available, although it has been shown that another substitution in this position (K601E) increases BRAF kinase activity [11]. The variants at D594 (the most frequently mutated residue in our patients, with D594N in four cases, D594G in three, and D594E in one) are more puzzling as they have been described as inactivating BRAF, making it unable to phosphorylate MEK, activate CRAF, or stimulate cell signalling. Interestingly, these variants are the third most common *BRAF* mutations in cancer (Cosmic Release v70), and often co-exist with *RAS* mutations [13], which otherwise generally occur in a mutually exclusive manner.

**Figure 2:**
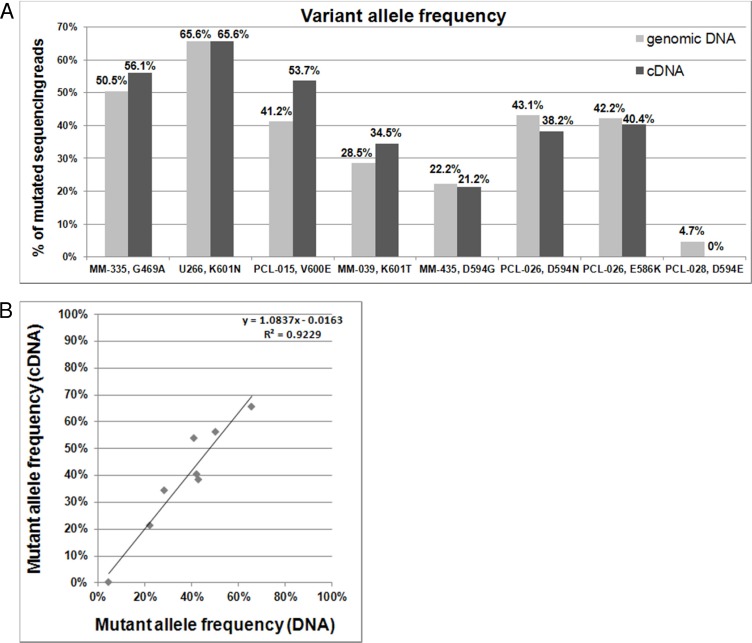
*BRAF* mutations detected on genomic DNA and cDNA **A**. Percentages of variant *BRAF* sequencing reads identified by NGS analyses of genomic DNA and retrotranscribed total RNA. **B**. Correlation between VAFs detected on genomic DNA and cDNA.

In order to assess whether the identified mutations were actually expressed, we sequenced those of our *BRAF*-mutated patients for whom mRNA material was available. Notably, the NGS results indicated that the VAFs detected in genomic DNA and retro-transcribed RNA were significantly linearly correlated (Figure 2).

### *NRAS* and *KRAS* mutations

In order to obtain a more complete picture of MEK-ERK signalling activation in MM, we also investigated the entire dataset for mutations in *NRAS* and *KRAS*, two GTPases that are known for long time to be involved in myelomagenesis [4, 6, 14, 15].

Sequencing of the mutation hot-spots of *NRAS* (exons 2 and 3) and *KRAS* (exons 2, 3 and 4) revealed the occurrence of mutations in respectively 23.9% (40/167) and 29.3% of the cases (49/167). As a whole, *NRAS* and *KRAS* mutations were detected in 55.3% of MM cases, 20.8% of pPCL, and 54.5% of sPCL.

In particular, *NRAS* mutations were found in 26.5% of the MMs at onset, 4.2% of the pPCLs, and 36.4% of the sPCLs (*P* = 0.008) (Supplementary Table 2A), whose main molecular features are shown in Supplementary Table 2B. In line with previous studies (particularly in MM), virtually all of the identified mutations affected hot-spot codons 61 (36/50, 72%), 13 (5/50, 10%) and 12 (3/50, 6.5%).

*KRAS* mutations were detected in 32.6% of the MMs at onset, 16.7% of the pPCLs, and 18.2% of the sPCLs; the most frequently targeted residues were Gly12 and Gln61, followed by Gly13 and other codons in exons 2, 3, and 4, each mutated in one or two cases (Supplementary Table 3A). The frequency of mutational substitution for a particular amino acid at these codons was absolutely consistent with previous observations in MM [4, 6].

The *NRAS* mutations showed only a weak negative association with *MAF*/*MAFB* translocations (*P* = 0.0775) (Supplementary Table 2C), and chromosome 17p deletions were less frequent in *KRAS*-mutated samples (*P* = 0.0019) (Supplementary Table 3B-C). The latter were also characterized by more frequent hyperdiploidy (*P* = 0.0124), although this association disappeared when the analysis was restricted to the MM samples alone (data not shown), probably because of the low prevalence of hyperdiploidy in the PCL samples.

**Figure 3:**
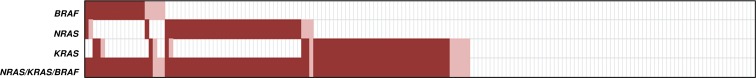
Heat map distribution of MAPK pathway gene mutations among MM/PCL patients The rows correspond to the indicated genes, and columns represent individual MM or PCL samples, which are colour-coded on the basis of gene status (white: wild-type; light red: Sanger-undetectable mutations; dark red: Sanger-detectable mutations).

Considering on the whole the three analyzed genes, the MEK/ERK signalling pathway was affected by mutational events in more than half of the cases (96/167, 57.5%) (Figure 3), being more frequent in sPCL (7/11, 63.6%) and MM (79/132, 59.8%), and relatively less frequent in pPCL (10/24, 41.7%); this latter finding is consistent with what newly emerged from a WES study in a smaller fraction of the pPCL patients of the present series [16]. Confirming recent data indicating multiple mutations within the same pathway [4, 6], we identified 13 samples with concomitant mutations in two genes (Figure 3): three cases in *BRAF* and *NRAS*, five in *BRAF* and *KRAS*, and five in *NRAS* and *KRAS*. In all patients, the co-existing mutations had different VAFs, thus supporting the occurrence of tumor subclones. Notably, five of the eight *BRAF* variants in D594 that are predicted to cause BRAF inactivation [13] co-existed with a *NRAS* or *KRAS* mutation.

Next, based on VAF, sample purity, and, if available, DNA copy number at *BRAF, NRAS* or *KRAS* genomic *loci*, we estimated the expected fraction of MM cells harboring each identified mutation. We then compared it with the percentage of CD138 positive-cells carrying main cytogenetic alterations (such as *IGH* translocations, hyperdiploidy, deletion of chromosome 13 or 1p, or 1q gain) as assessed by fluorescence *in situ* hybridization (data not shown). In such a way, we found that one of the following scenarios (observed at quite comparable frequencies in our series) occurred in most of the cases: (i) the same fraction of cells (corresponding to the whole tumor clone or to a part thereof) was affected both by mutations and other chromosomal lesions, or (ii) mutations occurred in a subclone of MM cells harboring other alterations. In line with recent WES studies, these data indicate that, although involving driver genes, MAPK mutations can be clonal (compatible with early events) in some patients or subclonal (compatible with late events) in others [4, 6], and may occur at variable times during tumor evolution compared to the other molecular lesions.

### Sequential analysis of *BRAF, NRAS* and *KRAS* mutations

**Figure 4:**
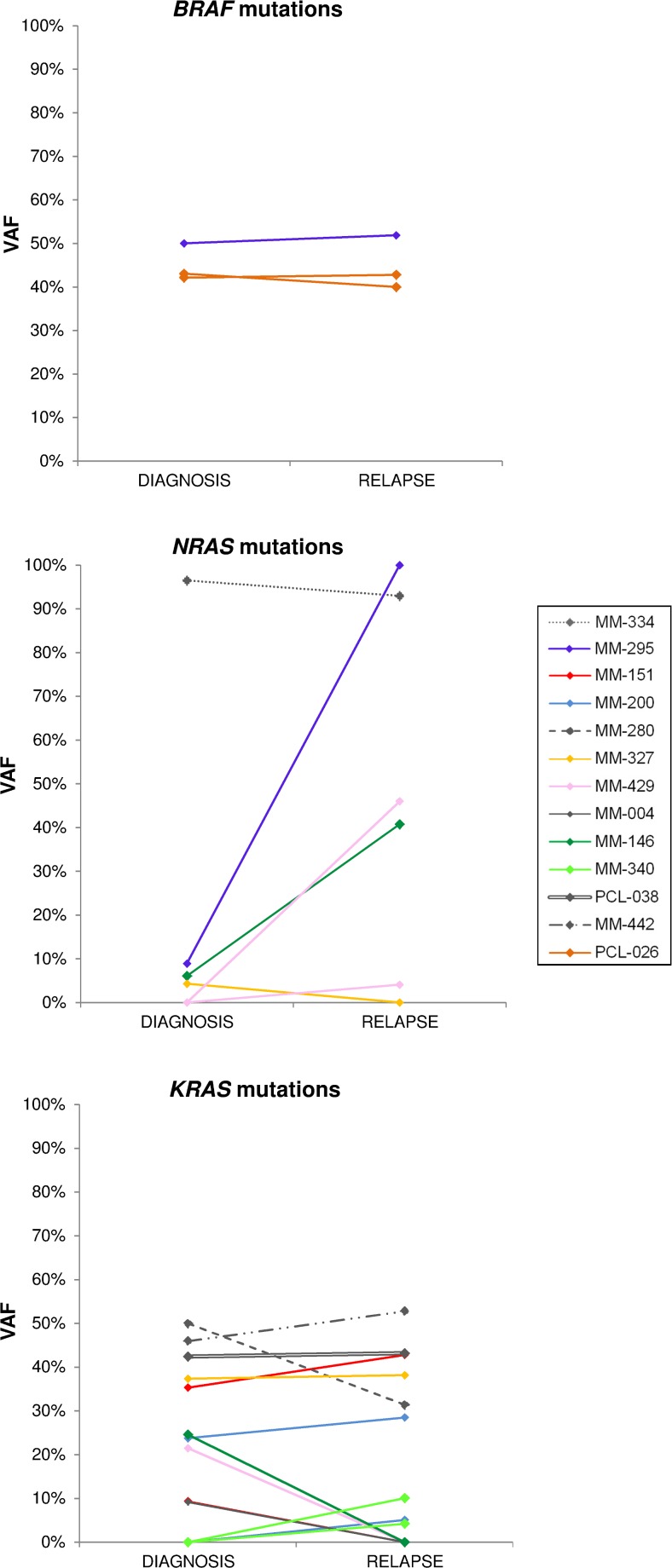
Changes of *BRAF, NRAS* and *KRAS* mutational burden during disease progression Allele frequencies of each variant are plotted at both time points for patients found mutated at diagnosis and/or relapse. The VAFs of mutations co-occurring in an individual patient are identified by the same color.

In order to gain further insights into the longitudinal status of *BRAF*/*NRAS*/*KRAS* mutations, we analyzed specimens taken from 19 patients at two different times: 14 with MM and two with pPCL at onset and relapse; two with MM at onset and at the time of leukemic transformation; and one at early and relapsed leukemic phase (Table 2 and Figure 4). The second specimens in each case were collected after at least one line of treatment with various regimens. In six cases (MM-239, MM-263, MM-271, MM-282, MM-286, and MM-281), all three genes were wild-type at both timepoints. Four of the remaining patients had one mutated gene (*BRAF* in PCL-026, *NRAS* in MM-334, and *KRAS* in PCL-038 and MM-442) at a quite constant load throughout disease course. Others showed the appearance or disappearance of *NRAS* and *KRAS* variants with low VAFs (the occurrence of *KRAS* G12A and G12R in MM-340; the occurrence of *KRAS* Q61L in MM-200, which also retained *KRAS* Q61H at a quite constant VAF; the loss of *KRAS* G12S in MM-004; the loss of *KRAS* G13D in MM-151, which also stably carried *KRAS* Q61H; the loss of *NRAS* Q61K in MM-327, which also harbored the *KRAS* Q61H mutation in about 40% of the sequencing reads at both timepoints). A reduction in the mutational load of a fully clonal variant (*KRAS* Q22K, with an allele frequency of 50% at MM onset and 31% at relapse) was found in one case (MM-280). Interestingly, the disappearance of a high frequency mutation was associated in all cases with the occurrence/clonal expansion of a further mutation in another gene of the pathway (the loss of *KRAS* G12D in MM-429, which acquired the *NRAS* Q61R mutation; and the loss of *KRAS* G12V in MM-146, which showed a concurrent increase in the allele frequency of *NRAS* Q61H from 8% to 41%). Notably, the leukemic transformation in MM-295 was accompanied by a dramatic increase in the VAF of *NRAS* G12D (9% to 100%), whereas the mutation burden of the co-existing *BRAF* D594N variant remained stable at about 50%.

**Table 2:** Mutation status of *BRAF, NRAS* and *KRAS* genes in 19 sequentially analyzed patients

Sample name	Disease stage	*BRAF* status	*KRAS* status	*NRAS* status
MM-004	MM onset	wild type	G12S, 9.18%	wild type
	MM relapse	wild type	wild type	wild type
MM-146	MM onset	wild type	G12V, 24.58%	Q61H, 6.11%
	MM relapse	wild type	wild type	Q61H, 40.78%
MM-151	MM onset	wild type	G13D, 9.35%; Q61H, 35.29%	wild type
	MM relapse	wild type	Q61H, 42.79%	wild type
MM-200	MM onset	wild type	Q61H, 23.74%	wild type
	MM relapse	wild type	Q61H, 28.49%; Q61L, 5.07%	wild type
MM-239	MM onset	wild type	wild type	wild type
	MM relapse	wild type	wild type	wild type
MM-263	MM onset	wild type	wild type	wild type
	MM relapse	wild type	wild type	wild type
MM-271	MM onset	wild type	wild type	wild type
	MM relapse	wild type	wild type	wild type
MM-280	MM onset	wild type	Q22K, 50%	wild type
	MM relapse	wild type	Q22K, 31.41%	wild type
MM-282	MM onset	wild type	wild type	wild type
	MM relapse	wild type	wild type	wild type
MM-286	MM onset	wild type	wild type	wild type
	MM relapse	wild type	wild type	wild type
MM-327	MM onset	wild type	Q61H, 37.34%	Q61K, 4.28%
	MM relapse	wild type	Q61H, 38.15%	wild type
MM-334	MM onset	wild type	wild type	G12D, 96.49%
	MM relapse	wild type	wild type	G12D, 92.94%
MM-340	MM onset	wild type	wild type	wild type
	MM relapse	wild type	G12A, 4.23%; G12R, 10.05%	wild type
MM-429	MM onset	wild type	G12D, 21.47%	wild type
	MM relapse	wild type	wild type	Q61R, 46%; E62Kfs*6, 4.09%
MM-295	MM onset	D594N, 50%	wild type	G12D, 8.89%
	MM leukemic transformation	D594N, 51.88%	wild type	G12D, 100%
MM-281	MM onset	wild type	wild type	wild type
	MM leukemic transformation	wild type	wild type	wild type
PCL-026	pPCL onset	D594N, 43.05%; E586K, 42.15%	wild type	wild type
	pPCL relapse	D594N, 40%; E586K, 42.8%	wild type	wild type
PCL-038	pPCL onset	wild type	G12R, 42.42%	wild type
	pPCL relapse	wild type	G12R, 43.17%	wild type
MM-442	MM leukemic transformation	wild type	Q61H, 45.93%	wild type
	sPCL relapse	wild type	Q61H, 52.75%	wild type

### Transcriptomic profiles of patients with *BRAF*/*NRAS*/*KRAS* mutations

In order to identify the transcriptional profiles related to *BRAF, NRAS* and *KRAS* mutations in primary tumors, we used microarray technology to investigate a large number (*n* = 142) of the samples analyzed by NGS. Assuming that alterations in a limited number of myeloma cells do not appreciably affect gene expression, we arbitrarily chose a lower VAF cut-off value of 20% for the supervised analysis of 60 wild-type and 68 mutated patients. They differentially expressed 86 genes (18 of which emerged at the highest stringency level) (Supplementary Table 4A): 27 up- and 59 down-regulated in the mutated cases. Interestingly, functional enrichment analysis revealed the involvement of a statistically significant fraction of modulated transcripts in MAPK signalling (*PRKD2, SPRED2, MAPKAPK2, CD300A, ARL6IP5, DUSP6, PPM1L, GRB2, LAMTOR3, SPRED1, LYN, EDN1, RASGRP1* and *ACVR1B*) at both biological process (GO:0000165, *q*-value=3.29E-03) and pathway level (198779 WikiPathway, *q*-value=1.33E-02) (Supplementary Table 4B).

**Figure 5:**
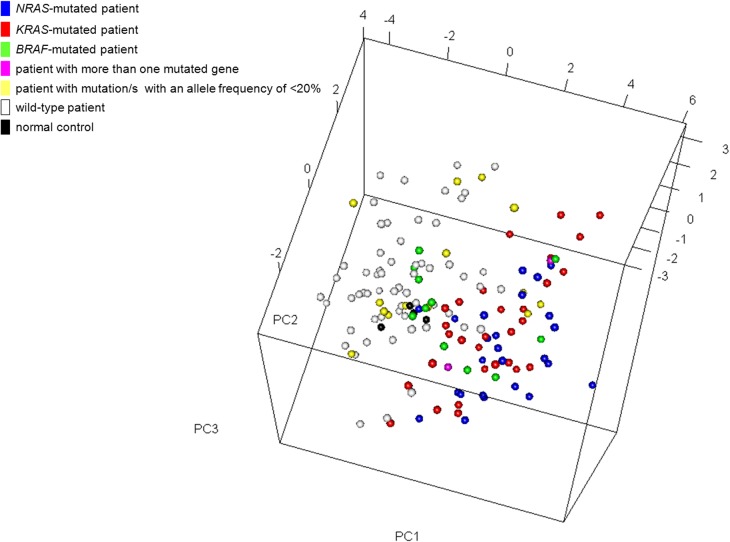
Multidimensional scaling plot using the most significant genes (*n* = 18) differentially expressed between *BRAF/NRAS/KRAS*-mutated and wild-type patients Each point represents a single sample and is coloured on its type (patient/normal control) and mutation status as measured by sequence analysis. In the case of co-existing mutations of which only one had an allele frequency of >20%, the color corresponds to the gene with the highest mutational load.

Principal component analysis (PCA) based on the expression of the 18 most statistically significant genes allowed a good separation between mutated and wild-type patients, without any apparent gene-specific discrimination (Figure 5). Notably, a few tumors not affected by mutations in any of the three genes (but possibly mutated in other genes of the pathway) showed the same activated MAPK pathway transcriptional profile as the mutated cases, as it has been found in other cancers [17]; conversely, some mutated samples had a wild-type-like expression profile, including 10 cases carrying mutations with a low VAF; MM-295 (*BRAF* D594N); and PCL-026, harboring both D594N and E586K *BRAF* mutations. Interestingly, NGS analysis of PCL-026 indicated that both variants were carried on the same allele, thus suggesting that the putative abrogation of BRAF negative regulation generated by the E586K substitution may not lead to increased BRAF signalling because of the concomitant occurrence of the D594N mutation.

### Molecular pathways dependent on BRAF-mediated signalling in MM

**Figure 6:**
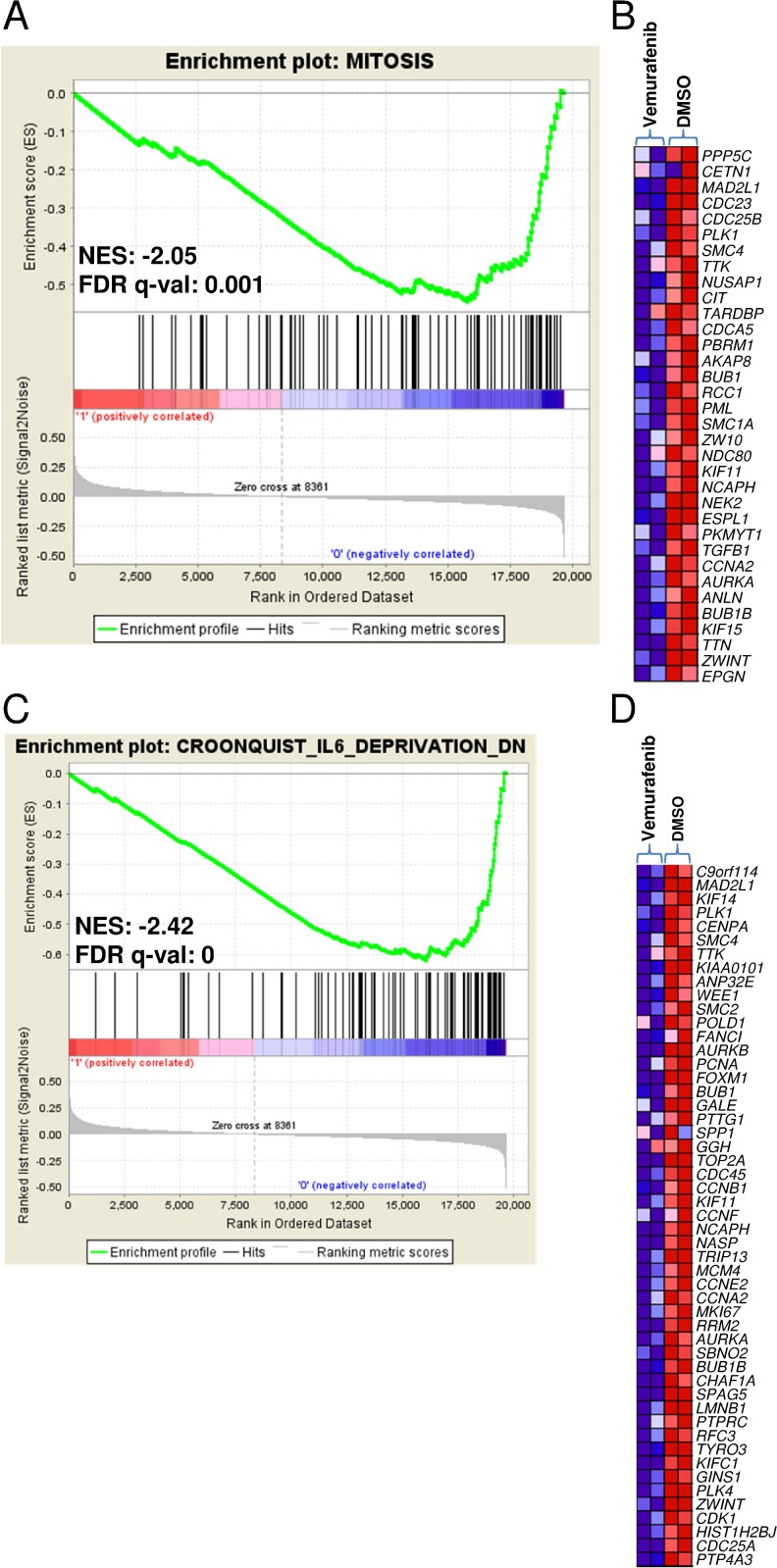
Enrichment plots and heat maps of selected gene sets detected by GSEA in U266 treated with vemurafenib in comparison with DMSO controls **A.**, **C.** The green curves show the enrichment score and reflect the degree to which each gene (black vertical lines) is represented at the bottom of the ranked gene list. Normalized enrichment score (NES) and FDRs are shown for each gene set; the gene sets with an FDR of <0.05 were considered enriched. **B.**, **D.** Heat maps of the genes constituting the leading edge subsets within the gene sets shown in panels A and C, respectively, in treated and control U266 cells.

In order to elucidate the transcriptional programmes related to BRAF activation in MM, we used vemurafenib (a BRAF inhibitor that has recently proved to be a promising anti-myeloma drug in clinical settings) [7] to inhibit BRAF activity in U266 cells which carry the K601N mutation and showing constitutive activation of MEK/ERK signalling (Supplementary Figure 2D). After confirming its ability to suppress the MAPK pathway and impair the proliferation of cultured U266 cells (Supplementary Figure 2A–2D), we compared the gene expression profiles of U266 cells treated with vemurafenib (30 μM) or DMSO for 12 hours. Gene set enrichment analysis (GSEA) was used to identify *a priori* defined sets of genes showing concordant modulation between treated and control phenotypes; in particular, the analysis ranked the genes on the basis of their differential expression between the classes (Supplementary Table 5A and B lists the 150 genes showing the highest up- and down-regulation following treatment) and identified a number of gene sets whose members tended to occur among genes with the largest differential expression between treated and control U266 cells (Supplementary Table 6A-B). The gene sets coordinately down-regulated in response to the drug included those associated with the gene ontology biological process of inactivating MAP kinase activity (Supplementary Figure 3A–3B) and mitosis (in line with the reduction of cell growth revealed by the cell viability analysis) (Figure 6A–6B). We also found other significantly down-regulated gene sets in the treated cells that are consistent with MEK-ERK pathway inhibition, including genes up-regulated in NIH3T3 cells transformed by activated *KRAS* [18]; genes down-regulated in the ANBL-6 MM cell line after *IL-6* withdrawal [19] (Figure 6C–6D) (also in line with the drug-induced down-regulation of *IL-6* in U266 cells) (Supplementary Table 5B); genes up-regulated in the proliferation (PR) subgroup of multiple myeloma described by Zhan *et al* [20] (Supplementary Figure 3C–3D); genes regulated by retinoblastoma via the E2F family of transcription factors; and genes identified in ovarian cancer downstream of CDKN1A and TP53 [21], which of note was up-regulated in the vemurafenib-treated U266 cells (Supplementary Table 5A). The gene sets that positively correlated with the treated phenotype (i.e. they were hyper-expressed following treatment) included those found up-regulated in MM1.S cells treated with the tyrosine kinase inhibitor adaphostin [22] (Supplementary Figure 4A–4B), and in response to the Ras-inhibitor salirasib in a panel of cancer cell lines with constitutively active HRAS [23] (Supplementary Figure 4C–4D).

## DISCUSSION

Members of the MAPK signalling pathway, such as *NRAS, KRAS* and *BRAF* are among the most frequently mutated proto-oncogenes in cancer. Notably, activating alterations of the *BRAF* serine/threonine kinase gene have also been recently described in whole-genome and exome sequencing (WGS/WES) studies of MM further extending the evidence of a widespread dysregulation of MAPK signalling in the disease [4-6, 24]. Over the last few years, *BRAF* has received considerable attention as a result of the success of targeted malignant melanoma therapy [25], and encouraging preliminary results have emerged from exploratory studies of the use of BRAF inhibitors in patients with *BRAF*-mutated MM [7, 9].

To the best of our knowledge, ours is the first study to use NGS to assess *BRAF, NRAS* and *KRAS* mutations in a large cohort of patients with MM (including primary and secondary leukemic forms) in the context of other clinical and biological features of the disease, and to determine a MAPK mutation-associated transcriptional signature in MM.

*BRAF* mutations were identified in 12% of the study population. Their frequency in our series of patients with MM at onset (10.6%) was more consistent with that found in WGS/WES studies [4, 6] than that found in other studies [7, 26]. Our NGS analysis identified mutations with a low VAF and a number of variants other than the classic V600E, the most common variant in melanoma and a hallmark of hairy cell leukemia. Published functional data indicate that most *BRAF* mutational events are responsible for the activation of MEK-ERK pathway, which is also caused by alterations in *NRAS* or *KRAS* (whose involvement in disease pathogenesis has already been extensively proven) [15, 26, 27] in an even more substantial percentage of patients.

We detected genetic alterations in ERK signalling in 57% of our patients, thus confirming the crucial role this pathway plays in MM development. In line with the findings of the most recent sequencing studies [4, 6], our analysis revealed concomitant substitutions in more than one of the investigated genes in 13 patients, although their simultaneous occurrence at high VAF was rare. Interestingly, the concomitant occurrence of five of the eight ^D594^ *BRAF* variants with mutations in *KRAS* or *NRAS* may be explained by the experimentally described synergy of kinase-dead *BRAF* mutations involving D594 and oncogenic *RAS.* Their cooperation in inducing tumor progression, indeed, has been demonstrated in a murine model of melanoma [13], thus confirming the hypothesis that the high frequency of this inactivating mutation (also observed in our MM patient cohort) is incompatible with a random event.

Furthermore, serial analyses of *BRAF, N*- and *K-RAS* mutations during disease progression revealed a slightly more complex scenario than that found by Bolli *et al*. [4], who described either clonal variants at both timepoints or the presence of acquired/increased variants in the later sample, in line with the expected positive selection of mutated subclones. Besides these patterns, we also observed the disappearance of variants with relatively high VAF values, but always occurring concurrently with the emergence/clonal expansion of an additional mutation in another gene of the pathway.

The activation of the MEK-ERK pathway as a result of *BRAF* and *RAS* mutations identified by NGS was confirmed by the GEP data of the same patients, indicating that the MAPK cascade is one of the most enriched biological processes involving genes that are differentially expressed between mutated and wild-type patients. Together with the detection of the majority of *BRAF* genomic variants at cDNA level, this highlights the particular importance of alterations in this pathway in terms of both occurrence and phenotypical impact in the context of the heterogeneous mutational pattern of MM. A recent RNA-seq analysis of a subset of patients who had previously undergone WES, indeed, has shown that most of the identified mutations occur in genes whose expression is very low or undetectable [28]; in addition, the mutation frequencies at genomic and transcriptional levels were found not comparable for many genes [28]. As far as our study is concerned, we observed that genomic and transcriptional VAFs of *BRAF* were highly concordant in mutated patients. This new finding further strengthens the rationale for *BRAF*-targeted therapeutic strategies. Furthermore, gene expression analysis of vemurafenib-treated *BRAF* mutated U266 cells revealed that the blockade of BRAF activation was accompanied by changes in the expression levels of critical genes for myeloma cells, such as *TP53*, or *IL*-6 and MM proliferation-associated genes [20] (respectively up- and down-regulated following treatment). These data are consistent with the findings from preclinical studies of MEK inhibitors, showing the inhibition of myeloma cell proliferation and the abrogation of paracrine signals for MM cell survival within the bone marrow niche, thus blocking osteoclast differentiation and reducing myeloma-induced angiogenesis [29-32].

The clinical relevance of *BRAF* and *NRAS/KRAS* mutations is still unclear. Bolli *et al*. found no signiﬁcant survival difference between cases with and without *KRAS, NRAS* or *BRAF* mutations [4], whereas Andrulis *et al*. [7] found that overall survival was significantly shorter in their MM patients carrying *BRAF* V600E. We were able to evaluate the impact of *KRAS, NRAS* or *BRAF* mutations in terms of progression-free survival, overall survival and response rates in our prospective series of pPCL patients followed up for 2.8 months but found no significant associations (data not shown). However, this was a small series and, as pPCL is a high-risk clinical entity with a complex pattern of genetic aberrations, it was not entirely suitable for such an assessment.

In conclusion, our data extend previous evidence that MEK-ERK pathway activation is very common in myeloma. The finding that it is the result of *BRAF* mutations in a significant percentage of patients has potentially immediate clinical implications. A few instances of the therapeutic use of the BRAF inhibitor vemurafenib in MM have been reported [7, 9], and it is to be hoped that more precise indications concerning its efficacy will emerge from the ongoing phase II Basket study (NCT01524978) of vemurafenib in patients with *BRAF* V600E mutation-positive cancers, which also includes MM patients [33]. However, the paradoxically tumor-enhancing effects of BRAF inhibitors in the case of sub-clonal *BRAF* or co-existent *BRAF* and *RAS* mutations indicate the need for the accurate molecular characterization of patients in order to obtain the most from targeted therapeutic strategies.

## MATERIALS AND METHODS

### Patients and cell lines

After the patients had given their informed consent in accordance with institutional guidelines (clearance from Ethic Committee, Fondazione Ospedale Maggiore di Milano), pathological BM specimens were obtained during standard diagnostic procedures from 132 patients with newly diagnosed MM, 24 cases of pPCL at onset and 11 cases of sPCL (79 males; median age 66 years, range 42-85), admitted from July 2001 to April 2014. MM and PCL were diagnosed based on the previously described criteria [2, 34]. One hundred patients had an immunoglobulin (Ig-)G protein monoclonal component; 34 IgA; one IgG/IgA; and one IgM protein; 101 cases had the light chain κ; 63 λ; two λ+κ. Twenty-five MM cases were in stage IA, 58 IIA/B and 49 IIIA/B, according to Durie and Salmon criteria [35]. Many of these patients have been described in previous papers [36, 37], and 16 of the 24 pPCL patients were participants in a multicentre clinical trial (RV-PCL-PI-350, EudraCT No. 2008-003246-28) [38].

The human myeloma cell lines (HMCLs) used in the study were NCI-H929, OPM2, JJN3, KMS-12, KMS-28, KMS-34, KMS-18, KMS-11, KMS-26, AMO1, RPMI 8226, delta-47, SK-MM-1, UTMC-2, MM.1S, U266, MM1-144, CMA-03 and CMA-03/06, LP-1, and KMS-27, all of which have been previously reported by us [39, 40] except for delta-47, UTMC-2, MM.1S and MM1-144 (kindly provided by Dr. G.Tonon - San Raffaele Scientific Institute, Milan).

### Sample preparation and molecular analyses

The BM specimens were collected from patients at the time of diagnosis; 19 cases were re-sampled at relapse/MM leukemic transformation after a median of 30 months. The PCs were puriﬁed (≥90% in all cases) using CD138 immunomagnetic microbeads as previously described [41, 42]. The main genomic aberrations (*IGH* translocations, hyperdiploidy, del(13q), del(17p), and 1q gain) were characterized by fluorescence *in situ* hybridization (FISH) in all cases as previously described [43] (Supplementary Table 7).

### Mutation analyses

NGS of the *BRAF* (exons 11 and 15), *NRAS* (exons 2 and 3) and *KRAS* (exons 2-4) mutation hotspots was performed from genomic DNA on the Genome Sequencer Junior instrument (Roche-454 Life Sciences, Penzberg, Germany) as previously described [44]. Details concerning the primer sequences and sequencing protocol are available in the Supplementary Methods. The sequencing reads were mapped to the reference sequence (RefSeq NG_007873.2 for *BRAF*, NG_007572.1 for *NRAS*, and NG_007524.1 for *KRAS*) and analyzed by means of Amplicon Variant Analyzer software (Roche).

The presence of non-synonymous variants was verified in an independent PCR product by means of conventional sequencing whenever the sensitivity of Sanger's method was consistent with variant allele frequency (VAF), or by means of an additional ultra-deep pyrosequencing run (median depth of coverage=1 402x) in the case of variants with a low VAF. In order to exclude germline variants, we sequenced the matched normal DNA when available, or consulted the Human dbSNP Database at NCBI (Build 142) (http://www.ncbi.nlm.nih.gov/snp). The occurrence of *BRAF* variants was also verified at transcriptional level (Supplementary Methods).

### Gene expression profiling and data analysis

The samples for gene expression profiling (GEP) were profiled using the GeneChip Human Gene 1.0 ST array (Affymetrix, Santa Clara, CA, USA) as previously described [37] (Supplementary Methods). Supervised analyses of the patient dataset were made using the Signiﬁcant Analysis of Microarrays (SAM) software [45]. The list of differentially expressed genes was submitted to the ToppGene Suite portal (http://toppgene.cchmc.org) for functional enrichment analysis using the ToppFun application [46]. For GEP of BRAF inhibitor-treated or untreated U266 cells (two independent replicas for each condition), microarray data were globally analyzed by Gene Set Enrichment Analysis (GSEA) [47]. Details concerning GEP data generation are given in the Supplementary Methods.

The principal component analysis (PCA) of the samples was performed by singular value decomposition of the considered data expression matrix using the *prcomp* function in the *stats* package, and the results were visualized using the *plot3d* function in the *rgl* package for R software.

The GEP data have been deposited in the NCBI Gene Expression Omnibus database (GEO; http//www.ncbi.nlm.nih.gov/geo; accession No. GSE66293).

### Statistical analysis

All of the contingency analyses were made using two-sided Fisher's exact test (*P* value < 0.05).

### Cell-based assays

The U266 cell line was maintained in RPMI 1640 medium supplemented with 10% fetal calf serum. Vemurafenib (Sigma-Aldrich, Saint Louis, MO, USA) was dissolved in dimethyl sulfoxide (DMSO) stock solutions and stored at −20°C before being added to log-phase cells at 30 μM on the basis of the findings of previous experiments testing dose-dependent cell proliferation and survival (data not shown), which confirmed the cells’ very weak sensitivity to the drug [48]. Standard procedures were used to assess the number and viability of the treated and control cells, and analyze the cell cycle and apoptosis (Supplementary Methods).

### Western blot analysis

Total cell extracts underwent standard SDS-PAGE and Western blotting procedures (see Supplementary Methods for antibody description).

## SUPPLEMENTARY MATERIAL TABLES AND FIGURES


