# Supplementary material for: Molecular spectrum of BRAF, NRAS and KRAS gene mutations in plasma cell dyscrasias: implication for MEK-ERK pathway activation
Source: Oncotarget. 2015 Jun 10;6(27):24205–17. doi: 10.18632/oncotarget.4434 (PMC4695180; doi:10.18632/oncotarget.4434)
Supplement: Supplementary file 1 [file oncotarget-06-24205-s001.pdf]

Molecular spectrum of *BRAF, NRAS* and *KRAS* gene mutations in plasma cell dyscrasias: implication for MEK-ERK pathway activation

Supplementary Material

Supplementary Materials and Methods……………………………………………2

Supplementary Tables…………………………………………………………….….7

Supplementary Figures…………………………………………………………..…36

Supplementary Materials and Methods

Mutation analyses

Genomic DNA was amplified using FastStart High Fidelity Polymerase (Roche) and fusion primers (Roche) containing M13 adapter sequences and the sequence-specific primers spanning *BRAF* exons 11 and 15 (RefSeq NM_004333.4) (exon 11 forward 5'-TCCCTCTCAGGCATAAGGTAA-3'; exon 11 reverse 5'-CGAACAGTGAATATTTCCTTTGAT-3'; exon 15 forward 5'-TCATAATGCTTGCTCTGATAGGA-3'; exon 15 reverse 5'-GGCCAAAAATTTAATCAGTGGA-3') [1], *NRAS* exons 2 and 3 (exon 2 forward 5'-ATGGAAGGTCACACTAGGGTT-3'; exon 2 reverse 5'-TCCTTTAATACAGAATATGGG-3'; exon 3 forward 5'-ACCCCCAGGATTCTTACAGA-3'; exon 3 reverse 5'-AGAGGTTAATATCCGCAAATG-3') [2], and *KRAS* exons 2, 3 and 4 (exon 2 forward 5'-TTAACCTTATGTGTGACATGTTCTAA-3'; exon 2 reverse 5'-GAAAGTAAAGTTCCCATATTAATGGT-3'; exon 3 forward 5'-GCACTGTAATAATCCAGACT-3'; exon 3 reverse 5'-CATGGCATTAGCAAAGACTC-3' [3]; exon 4 forward 5'-AGTTGTGGACAGGTTTTGAAAGA-3'; exon 4 reverse: 5'-ATTTCAGTGTTACTTACCTGTCTTG-3' (Primer-BLAST at http://www.ncbi.nlm.nih.gov/tools/primer-blast/index.cgi?LINK_LOC=BlastHome)). Amplicon library A and B sequencing adapters and multiplex identifier (MID) tags were then added to both tails of amplicons by a second amplification step. PCR conditions were as follows: in the first amplification step, denaturating step at 94°C for 5 min followed by 25 cycles at 94°C (30 sec. per cycle), annealing step at 57°C (30 sec. per cycle), and extension at 72°C (45 sec. per cycle), followed by a final 7 min. extension at 72°C; in the second amplification step, denaturating step at 94°C for 5 min followed by 25 cycles at 94°C (20 sec. per cycle), annealing step at 55°C (20 sec. per cycle), and extension at 72°C (45 sec. per cycle), followed by a final 10 min. extension at 72°C. All PCR samples were purified using an AMPure XP (Agencourt) bead clean-up; samples were then quantitated using picogreen dye (Life Technologies) and the Victor X2 (Perkin Elmer) fluorometer. Samples were pooled together at equimolar ratios to prepare for Roche/454 pyrosequencing. The obtained amplicon library was added to the emulsion PCR at a ratio of 0.8 molecules per bead and subjected to deep sequencing on the Genome Sequencer Junior instrument (Roche-454 Life Sciences). The obtained sequencing reads were mapped to the reference sequence (RefSeq NG_007873.2 for *BRAF*, RefSeq NG_007572.1 for *NRAS*, RefSeq NG_007524.1 for *KRAS*) and analyzed by the Amplicon Variant Analyzer software (Roche) to establish the mutant allele frequency.

To verify the occurrence of *BRAF* variants at transcriptional level, total RNA of mutated samples was converted to cDNA using M-MLV reverse transcriptase (Invitrogen) and random hexamers, and subjected to deep sequencing of the exon harboring the variant/s detected on genomic DNA. Sequence-specific exonic PCR primers were designed in the Primer 3 program (http://frodo.wi.mit.edu/primer3/) and are as follows: exon 11 forward 5'-CTCAGCGAGAAAGGAAGTCA-3'; exon 11 reverse 5'-GGAATAGCCCATGAAGAGTAGGA-3'; exon 15 forward 5'-AGATATTGCACGACAGACTGC-3'; exon 15 reverse 5'-TTCTGATGACTTCTGGTGCC-3'.

Gene expression profiling and data analysis

For gene expression analysis, samples were profiled on the GeneChip Human Gene 1.0 ST array (Affymetrix, Santa Clara, CA, USA) as previously described [4]. The raw intensity expression values were processed by Robust Multi-array Average procedure (RMA) [5], with the re-annotated Chip Definition Files from BrainArray libraries version 18.0.0 [6], available at http://brainarray.mbni.med.umich.edu. Sno/scaRNA expression profiles were excluded in the following supervised analyses by Significant Analysis of Microarrays (SAM). In the patients' dataset, supervised analyses on gene expression profiles were carried out using the SAM software version 4.00 (excel front-end publicly available at http://www-stat.stanford.edu/ B tibs/SAM/index.html) [7]. The cutoff point for statistical significance (at a *q*-value = 0) was determined by tuning the Δ parameter on the false discovery rate (FDR) and controlling the *q*-value of the selected gene lists. A higher stringency level (90th percentile FDR = 0) was also applied to the differentially expressed gene lists at *q*-value 0. The list of differentially expressed genes was submitted to ToppGene Suite portal (http://toppgene.cchmc.org) for functional enrichment analysis by using ToppFun application [8]. A *P*-value cut-off of 0.05 and FDR Benjamini and Hochberg correction method were applied to all the annotation terms in the default parameter set, and those with a *q*-value < 0.05 were defined as significantly enriched. For gene expression analysis of U266 cells treated with BRAF-inhibitor or untreated (two independent replicas for each condition), microarray data were globally analyzed by Gene Set Enrichment Analysis (GSEA) [9] by means of gene sets of the MSigDB database (http://www.broadinstitute.org/gsea/ msigdb/index.jsp) consisting of 5-1 000 genes (C1 Positional and C4 Computational gene sets were not considered). Gene set permutations (1 000) were set and default parameters were applied for statistical analyses. Gene sets showing a false discovery rate lower than 5% were selected as significantly enriched in U266 treated and control phenotypes, respectively.

The principal component analysis of the samples was performed by singular value decomposition of the considered data expression matrix using *prcomp* function in *stats* package and visualized with *plot3d* function in *rgl* package for R software.

Cell lines

Details regarding the source of all cell lines have been previously reported by us [10, 11], except for delta-47, UTMC-2, MM.1S and MM1-144, that were kindly provided by Dr. G.Tonon - San Raffaele Scientific Institute, Milan. All cell lines have been recently authenticated by STR profiling and/or gene mutational analyses, and tested for mycoplasma contamination.

Cell-based assays

U266 cell line was maintained in RPMI 1640 Medium supplemented with 10% fetal calf serum. Vemurafenib (Sigma-Aldrich) was dissolved in dimethyl sulfoxide (DMSO) stock solutions and stored at -20°C. The drug was added to log phase cells and used at 30 μM, based on previuos experiments testing cell proliferation and survival in a dose-dependent manner (data not shown) that confirmed the very weak sensitivity of U266 to the drug [12].
For proliferation assays, U266 cells were plated in 6 or 96-well plates, at 0.5 x 10^6 or 0.1 x 10^6 cells/well respectively and were treated with Vemurafenib or vehicle (DMSO) as control for 6, 12, 24, 48, 72 hrs. Cell number and viability were assessed by Trypan Blue exclusion and cell proliferation was evaluated by Thiazolyl Blue Tetrazolium Bromide (MTT) assay (Sigma Aldrich, USA), following the manufacturer's instructions.
For cell cycle analysis, cells were then collected after 6, 12, 24, 48, 72 h, fixed in 70% ethanol, and stored at 4°C until analysis. Before cytofluorimetric analysis, 10^6 cells were washed twice in PBS and incubated in staining solution (10 mg/ml propidium iodide, 100 mg/ml RNase) at room temperature in the dark for at least 2 h. Cell cycle profiles were assessed using a FACSCalibur device (Becton Dickinson, San Jose´, CA, USA) and analyzed using Multicycle AV™ software (version 4; De Novo Software). For apoptosis analysis, cells were harvested after 6, 12, 24, 48, 72 h and treated with Annexin V/7-aminoactinomycin D (7-AAD) solution (BD Pharmingen) according to the manufacturer's instructions. The fluorescent intensity of at least 20 000 cells from each sample was measured. Data were acquired using CellQuest software (BD, San Jose, CA) and analyzed using FCS Express software (version 4, De Novo Software).

Western Blot Analysis

Standard SDS-PAGE and Western blotting procedures were applied to total cell extracts. Anti-BRAF (F7) antibody (catalog no.sc-5284) was purchased from Santa Cruz

Biotechnology; anti-phospho-ERK1/2 (product no. M8159), anti-ERK1/2 (product no.M5670) and anti-B actin (product no. A2066) antibodies were purchased from Sigma-Aldrich.

References

1. Davies H, Bignell GR, Cox C, Stephens P, Edkins S, Clegg S, Teague J, Woffendin H, Garnett MJ, Bottomley W, Davis N, Dicks E, Ewing R, *et al.* Mutations of the BRAF gene in human cancer. Nature. 2002; 417:949-954.
2. Chng WJ, Gonzalez-Paz N, Price-Troska T, Jacobus S, Rajkumar SV, Oken MM, Kyle RA, Henderson KJ, Van Wier S, Greipp P, Van Ness B, Fonseca R. Clinical and biological significance of RAS mutations in multiple myeloma. Leukemia. 2008; 22:2280-2284.
3. Chang YS, Yeh KT, Hsu NC, Lin SH, Chang TJ, Chang JG. Detection of N-, H-, and KRAS codons 12, 13, and 61 mutations with universal RAS primer multiplex PCR and N-, H-, and KRAS-specific primer extension. Clinical biochemistry. 2010; 43:296-301.
4. Todoerti K, Agnelli L, Fabris S, Lionetti M, Tuana G, Mosca L, Lombardi L, Grieco V, Bianchino G, D'Auria F, Statuto T, Mazzoccoli C, De Luca L, *et al.* Transcriptional characterization of a prospective series of primary plasma cell leukemia revealed signatures associated with tumor progression and poorer outcome. Clinical cancer research : an official journal of the American Association for Cancer Research. 2013; 19:3247-3258.
5. Irizarry RA, Hobbs B, Collin F, Beazer-Barclay YD, Antonellis KJ, Scherf U, Speed TP. Exploration, normalization, and summaries of high density oligonucleotide array probe level data. Biostatistics. 2003; 4:249-264.
6. Dai MH, Wang PL, Boyd AD, Kostov G, Athey B, Jones EG, Bunney WE, Myers RM, Speed TP, Akil H, Watson SJ, Meng F. Evolving gene/transcript definitions significantly alter the interpretation of GeneChip data. Nucleic acids research. 2005; 33:e175.
7. Tusher VG, Tibshirani R, Chu G. Significance analysis of microarrays applied to the ionizing radiation response. Proceedings of the National Academy of Sciences of the United States of America. 2001; 98:5116-5121.
8. Chen J, Bardes EE, Aronow BJ, Jegga AG. ToppGene Suite for gene list enrichment analysis and candidate gene prioritization. Nucleic acids research. 2009; 37:W305-311.

9. Subramanian A, Tamayo P, Mootha VK, Mukherjee S, Ebert BL, Gillette MA, Paulovich A, Pomeroy SL, Golub TR, Lander ES, Mesirov JP. Gene set enrichment analysis: a knowledge-based approach for interpreting genome-wide expression profiles. Proceedings of the National Academy of Sciences of the United States of America. 2005; 102:15545-15550.

10. Lombardi L, Poretti G, Mattioli M, Fabris S, Agnelli L, Bicciato S, Kwee I, Rinaldi A, Ronchetti D, Verdelli D, Lambertenghi-Deliliers G, Bertoni F, Neri A. Molecular characterization of human multiple myeloma cell lines by integrative genomics: Insights into the biology of the disease. Gene Chromosome Canc. 2007; 46:226-238.

11. Verdelli D, Nobili L, Todoerti K, Mosca L, Fabris S, D'Anca M, Pellegrino E, Piva R, Inghirami G, Capelli C, Introna M, Baldini L, Chiaramonte R*, et al.* Molecular events underlying interleukin-6 independence in a subclone of the CMA-03 multiple myeloma cell line. Genes, chromosomes & cancer. 2014; 53:154-167.

12. Lode L, Moreau P, Menard A, Godon C, Touzeau C, Amiot M, Le Gouill S, Bene MC, Pellat-Deceunynck C. Lack of BRAF V600E mutation in human myeloma cell lines established from myeloma patients with extramedullary disease. Blood Cancer J. 2013; 3:e163.

Supplementary Table 1A: Clinical and molecular characteristics of 20 MM/PCL patients carrying non-synonymous *BRAF* mutations

Sample name	*Disease*	*Variant (GRCh38)*	*% of mutated sequencing reads*	*AA change*	*Sex*	*Age*	*PP*†	*del(13)**	*del(17p)**	*1q gain**	*1p loss**	*t(4;14)*	*t(11;14)*	*t(14;16)*	*t(14;20)*	*HD*°
**MM-036**	MM	140753355C>T	5.61%	D594N	M	65	Gκ	+	-	+	nd	-	-	-	-	-
**MM-039**	MM	140753333T>G	28.53%	K601T	M	50	Gλ	-	-	-	nd	-	-	-	-	+
**MM-140**	MM	140753355C>T	1.68%	D594N	M	62	na	-	-	-	nd	-	+	-	-	-
**MM-177**	MM	140753336A>T	27.18%	V600E	M	73	Gκ	+	-	+	-	-	-	-	-	-
**MM-219**	MM	140753354T>C	7.78%	D594G	M	73	Aλ	+	-	-	-	-	-	-	-	+
**MM-224**	MM	140753349C>G	35.44%	G596R	F	52	Gκ	-	-	+	-	-	-	+	-	-
**MM-268**	MM	140753336A>T	29.57%	V600E	M	77	Gκ	-	-	-	-	-	-	-	-	-
**MM-295**	MM	140753355C>T	50.00%	D594N	F	74	λ	+	-	nd	nd	-	-	-	-	nd
**MM-313**	MM	140753336A>T	44.59%	V600E	M	66	Gλ	-	-	-	-	-	+	-	-	-
**MM-335**	MM	140781602C>G	50.52%	G469A	F	68	Aκ	+	-	-	-	-	-	+	-	-
**MM-411**	MM	140753354T>C	0.86%	D594G	M	67	Gλ	-	-	-	-	-	-	-	-	+
**MM-435**	MM	140753354T>C	22.22%	D594G	na	42	Gλ	-	-	-	-	-	-	-	-	+
**MM-441**	MM	140753336A>T	35.74%	V600E	na	na	Gλ	-	-	-	-	-	-	-	-	-
**MM-446**	MM	140753336A>T	26.48%	V600E	na	52	Gλ	-	-	+	-	-	-	-	-	+
**PCL-015**	pPCL	140753336A>T	41.18%	V600E	M	78	k	+	-	+	-	-	-	+	-	-
**PCL-023**	pPCL	140781602C>G	70.70%	G469A	M	60	Gκ	+	+	+	+	-	-	+	-	-
**PCL-026**	pPCL	140753355C>T<br>140753379C>T	43.05%<br>42.15%	D594N<br>E586K	F	59	Gκ	+	-	+	-	-	-	+	-	-
**PCL-028**	pPCL	140753353A>C	4.65%	D594E	F	57	κ	+	-	-	-	-	+	-	-	-
**PCL-042**	sPCL	140781602C>G	51.26%	G469A	F	69	Gλ	-	-	-	-	-	+	-	-	-
**PCL-043**	pPCL	140753336A>T	44.59%	V600E	F	68	Gλ	-	-	-	-	-	+	-	-	-

†Paraprotein; *del(13), del(17), 1p loss and 1q gain were determined by FISH. °HD = presence of the hyperdiploid status on the basis of FISH evaluation criteria.

Supplementary Table 1B: Clinical and molecular characteristics of the 167 MM/PCL patients analyzed for *BRAF* mutations

Characteristic	*All patients (n=167)*		*BRAF wild type (n=147)*		*BRAF mutated (n=20)*		*P value*[a]
	N	*%*	*n*	*%*	*n*	*%*	
MM	132	*79*	118	*80.3*	14	*70*	
pPCL	24	*14.4*	19	*12.9*	5	*25*	n.s.
sPCL	11	*6.6*	10	*6.8*	1	*5*	
del(13q)	79	*47.6*	70	*47.9*	9	*45*	n.s.
chr 13 disomic patients	87	*52.4*	76	*52.1*	11	*55*	
del(17p)	18	*10.9*	17	*11.7*	1	*5*	n.s.
17p disomic patients	147	*89.1*	128	*88.3*	19	*95*	
1q gain	68	*43*	61	*43.9*	7	*36.8*	n.s.
1q disomic patients	90	*57*	78	*56.1*	12	*63.2*	
1p loss	18	*12.7*	17	*13.5*	1	*6.25*	n.s.
1p disomic patients	124	*87.3*	109	*86.5*	15	*93.75*	
IGH trx	82	*49.7*	72	*49.6*	10	*50*	n.s.
no *IGH* trx	83	*50.3*	73	*50.4*	10	*50*	
hyperdiploid	50	*32.9*	45	*33.8*	5	*26.3*	n.s.
non-hyperdiploid	102	*67.1*	88	*66.2*	14	*73.7*	

[a] Significance was assessed by Freeman-Halton extension of Fisher's exact test for disease type, and by Fisher's exact test for all other variables.

Supplementary Table 2A: Non-synonymous *NRAS* mutations identified by NGS in MM/PCL patients

Variant (GRCh38)	*AA change*	*dbSNP ID*	*COSMIC ID (v71)*	*MM literature**	*Mutated patients (percentage of mutated sequencing reads)*												
114713908T>C	Q61R	rs11554290	COSM584	1, 2, 3	MM-424 (95.95%)	PCL-041 (90.50%)	MM-174 (80.77%)	PCL-039 (63.37%)	MM-212 (46.70%)	MM-034 (46.37%)	MM-049 (34.44%)	MM-435 (29.87%)	MM-241 (28.98%)	MM-381 (21.99%)	MM-261 (21.63%)	MM-229 (11.68%)	MM-387 (4.56%)
114713909G>T	Q61K	rs121913254	COSM580	1, 2, 3, 4	MM-434 (88.27%)	MM-449 (84.36%)	PCL-005 (45.05%)	MM-246 (40.98%)	MM-330 (33.91%)	MM-414 (31.87%)	MM-087 (31.74%)	MM-448 (26.60%)	MM-037 (12.22%)	PCL-039 (8.26%)	MM-327 (4.28%)	MM-284 (5.86%)	
114713907T>G	Q61H	/	COSM586	1, 3, 4	MM-398 (47.45%)	MM-405 (43.42%)	MM-341 (31.48%)	MM-387 (18.46%)	PCL-027 (65.62%)	MM-302 (15.73%)							
114716124C>G	G13R	rs121434595	COSM569	1, 3, 4	MM-372 (52.60%)	MM-066 (30.19%)	MM-302 (17.44%)	MM-284 (4.61%)									
114716126C>T	G12D	rs121913237	COSM564	3	MM-334 (96.49%)	MM-422 (11.95%)	MM-295 (8.89%)										
114713907delT	E62Kfs*6	/	/	/	MM-405 (2.67%)	MM-212 (1.28%)	MM-229 (1.27%)										
114713907T>A	Q61H	rs121913255	COSM585	1, 3, 4	MM-317 (48.82%)	MM-146 (6.11%)											
114713908T>A	Q61L	rs11554290	COSM583	3	MM-276 (45.41%)	MM-448 (10.37%)											
114716123C>T	G13D	rs121434596	COSM573	3	PCL-011 (93.06%)												
114713908_114713909TG>GT	Q61T	/	/	/	MM-314 (82%)												
114713878T>C	Y71C	/	/	/	MM-276 (45.95%)												
114713831T>A	S87C	/	/	/	MM-411 (45.45%)												
114713831delT	S87Afs*17	/	/	/	MM-411 (2.24%)												

*1: Chapman *et al.*, Nature 2011; 2: Walker *et al.*, Blood 2012; 3: Lohr *et al.*, Cancer Cell 2014; 4: Bolli *et al.*, Nature Communications 2014.

Supplementary Table 2B: Clinical and molecular characteristics of 40 MM/PCL patients carrying non-synonymous *NRAS* mutations

Sample name	*Disease*	*Variant (GRCh38)*	*% of mutated sequencing reads*	*AA change*	*Sex*	*Age*	*PP*[†]	*del(13)**	*del(17p)**	*1q gain**	*1p loss**	*t(4;14)*	*t(11;14)*	*t(14;16)*	*t(14;20)*	*HD°*
MM-405	MM	114713907T>G/g.114713907delT	43.42%/2.67%	Q61H/E62Kfs*6	M	69	Gκ	-	-	-	-	-	+	-	-	-
MM-034	MM	114713908T>C	46.37%	Q61R	M	71	Gκ	-	-	-	-	-	-	-	-	+
MM-037	MM	114713909G>T	12.22%	Q61K	F	50	Gκ,Aκ	+	-	-	-	-	+	-	-	-
MM-049	MM	114713908T>C	34.44%	Q61R	M	62	κ	-	-	-	-	-	-	-	-	+
MM-066	MM	114716124C>G	30.19%	G13R	F	77	Aκ	-	-	-	na	+	-	-	-	-
MM-087	MM	114713909G>T	31.74%	Q61K	F	84	Gλ	+	-	+	na	+	-	-	-	-
MM-146	MM	114713907T>A	6.11%	Q61H	M	68	Gλ	-	-	+	na	-	-	-	-	+
MM-174	MM	114713908T>C	80.77%	Q61R	M	85	Aκ	-	-	-	-	-	-	-	-	+
MM-212	MM	114713908T>C/g.114713907delT	46.70%/1.28%	Q61R/E62Kfs*6	F	55	Gκ	-	-	-	-	-	+	-	-	-
MM-229	MM	114713908T>C/g.114713907delT	11.68%/1.27%	Q61R/E62Kfs*6	M	75	Gκ	-	-	-	-	-	-	-	-	-
MM-241	MM	114713908T>C	28.98%	Q61R	M	54	Gκ	-	-	-	-	-	-	-	-	+
MM-246	MM	114713909G>T	40.98%	Q61K	M	71	Aλ	-	-	-	-	-	+	-	-	-
MM-261	MM	114713908T>C	21.63%	Q61R	F	66	Gκ	+	-	+	-	-	-	-	-	+
MM-276	MM	114713878T>C/g.114713908T>A	45.95%/45.41%	Y71C/Q61L	F	70	Gκ	+	-	-	-	+	-	-	-	-
MM-284	MM	114716124C>G/g.114713909G>T	4.61%/5.86%	G13R/Q61K	M	49	k	na	-	-	-	-	+	-	-	-
MM-295	MM	114716126C>T	8.89%	G12D	F	74	λ	+	-	na	na	-	-	-	-	na
MM-302	MM	114716124C>G/g.114713907T>G	17.44%/15.73%	G13R/Q61H	M	65	Aκ	-	-	+	-	-	-	-	-	+
MM-314	MM	114713908_114713909TG>GT	82.04%	Q61T	F	70	Aλ	-	-	+	-	-	+	-	-	-
MM-317	MM	114713907T>A	48.82%	Q61H	M	56	Gλ	-	-	+	-	-	+	-	-	-
MM-327	MM	114713909G>T	4.28%	Q61K	F	62	absent	+	-	+	-	-	-	-	-	+
MM-330	MM	114713909G>T	33.91%	Q61K	F	61	Aκ	+	-	+	-	+	-	-	-	-
MM-334	MM	114716126C>T	96.49%	G12D	F	45	Gλ	+	+	+	+	-	-	-	-	+
MM-341	MM	114713907T>G	31.48%	Q61H	M	65	Gκ	-	-	+	-	-	-	-	-	-
MM-372	MM	114716124C>G	52.60%	G13R	M	54	Gk	-	-	+	-	-	+	-	-	-
MM-381	MM	114713908T>C	21.99%	Q61R	F	69	Gκ	-	-	-	-	-	-	-	na	na
MM-387	MM	114713907T>G/g.114713908T>C	18.46%/4.56%	Q61H/Q61R	F	44	Gκ	-	-	+	-	-	-	-	-	+
MM-398	MM	114713907T>G	47.45%	Q61H	F	65	Gκ	+	-	+	-	-	+	-	-	-
MM-411	MM	114713831T>A/g.114713831delT	45.45%/2.24%	S87C/ S87Afs*17	M	67	Gλ	-	-	-	-	-	-	-	-	+
MM-414	MM	114713909G>T	31.87%	Q61K	F	67	Gλ	-	-	-	-	-	+	-	-	-
MM-422	MM	114716126C>T	11.95%	G12D	F	74	k	+	-	-	-	-	-	-	-	-
MM-424	MM	114713908T>C	95.95%	Q61R	F	84	Gκ	+	-	-	+	-	-	-	-	+
MM-434	MM	114713909G>T	88.27%	Q61K	F	53	κ	+	-	-	+	-	-	-	-	-
MM-435	MM	114713908T>C	29.87%	Q61R	na	42	Gλ	-	-	-	-	-	-	-	-	+
MM-448	MM	114713909G>T/g.114713908T>A	26.60%/10.37%	Q61K/Q61L	na	59	Aκ	+	-	+	-	-	-	-	-	-
MM-449	MM	114713909G>T	84.36%	Q61K	na	54	Gκ	+	-	-	+	-	-	-	-	-
PCL-005	sPCL	114713909G>T	45.05%	Q61K	M	76	Aκ	-	-	-	-	-	+	-	-	-
PCL-011	sPCL	114716123C>T	93.06%	G13D	M	76	Gκ	+	+	+	-	-	-	+	-	-
PCL-027	pPCL	114713907T>G	65.62%	Q61H	M	65	λ	-	+	-	-	-	+	-	-	-
PCL-039	sPCL	114713908T>C/g.114713909G>T	63.37%/8.26%	Q61R/Q61K	M	54	Aκ	-	+	-	+	-	-	-	-	-
PCL-041	sPCL	114713908T>C	90.50%	Q61R	M	na	Gκ	-	-	na	na	-	na	na	na	na

[†]Paraprotein; *del(13), del(17), 1p loss and 1q gain were determined by FISH. °HD = presence of the hyperdiploid status on the basis of FISH evaluation criteria.

Supplementary Table 2C: Clinical and molecular characteristics of the 167 MM/PCL patients analyzed for *NRAS* mutations

Characteristic	*All patients (n=167)*		*NRAS wild type (n=127)*		*NRAS mutated (n=40)*		*P value*[a]
	N	*%*	*n*	*%*	*n*	*%*	
MM	132	*79*	97	*76.4*	35	*87.5*	
pPCL	24	*14.4*	23	*18.1*	1	*2.5*	0.008
sPCL	11	*6.6*	7	*5.5*	4	*10*	
del(13q)	79	*47.6*	64	*50.4*	15	*38.5*	n.s.
chr 13 disomic patients	87	*52.4*	63	*49.6*	24	*61.5*	
del(17p)	18	*10.9*	14	*11.2*	4	*10*	n.s.
17p disomic patients	147	*89.1*	111	*88.8*	36	*90*	
1q gain	68	*43*	53	*44.2*	15	*39.5*	n.s.
1q disomic patients	90	*57*	*67*	*55.8*	23	*60.5*	
1p loss	18	*12.7*	13	*12.1*	5	*14.3*	n.s.
1p disomic patients	124	*87.3*	94	*87.9*	30	*85.7*	
IGH trx	82	*49.7*	64	*49.8*	18	*46.1*	n.s.
no *IGH* trx	83	*50.3*	62	*50.2*	21	*53.8*	
hyperdiploid	50	*32.9*	37	*32.2*	13	*35.1*	n.s.
non-hyperdiploid	102	*67.1*	78	*67.8*	24	*64.9*	

[a] Significance was assessed by Freeman-Halton extension of Fisher's exact test for disease type, and by Fisher’s exact test for all other variables.

Supplementary Table 3A: Non-synonymous *KRAS* mutations identified by NGS in MM/PCL patients

Variant (GRCh38)	*AA change*	*dbSNP ID*	*COSMIC ID (v71)*	*MM literature**	*Mutated patients (percentage of mutated sequencing reads)*								
25227341T>G	Q61H	rs17851045	COSM554	1, 2, 3, 4	MM-055 (52.53%)	MM-442 (45.93%)	MM-430 (39.77%)	MM-440 (38%)	MM-327 (37.34%)	MM-202 (35.54%)	MM-151 (35.29%)	MM-402 (34.62%)	MM-200 (23.74%)
					MM-229 (12.07%)	MM-381 (9.51%)	MM-441 (8.45%)	PCL-046 (7.73%)	MM-300 (7.61%)				
25245350C>T	G12D	rs121913529	COSM521	3	MM-154 (45.28%)	MM-030 (45%)	MM-152 (31.69%)	MM-433 (31.78%)	MM-026 (30.59%)	MM-429 (21.47%)	PCL-046 (9.01%)		
25245351C>G	G12R	rs121913530	COSM518	2, 3, 4	MM-039 (46.94%)	PCL-038 (42.42%)	MM-445 (28.85%)	MM-159 (27.78%)					
25245347C>T	G13D	rs112445441	COSM532	1, 2, 3	MM-385 (40.68%)	MM-308 (39.42%)	MM-151 (9.35%)	MM-219 (5.88%)					
25227342T>C	Q61R	rs121913240	COSM552	2, 3, 4	MM-038 (40.82%)	MM-351 (28.66%)	MM-159 (3.75%)						
25245321G>T	Q22K	rs121913236	COSM543	4	MM-280 (50%)	MM-026 (32.94%)							
25225628C>T	A146T	rs121913527	COSM19404	1	MM-413 (51.24%)	MM-131 (40.52%)							
25245348C>A	G13C	rs121913535	COSM527	/	PCL-001 (48.64%)	MM-149 (29.13%)							
25225713T>G	K117N	/	COSM19940	1, 3	MM-310 (47.09%)	MM-269 (3.07%)							
25245328C>G	L19F	/	COSM12703	1	MM-386 (42.73%)	MM-335 (18.6%)							
25245350C>A	G12V	rs121913529	COSM520	3, 4	MM-146 (24.58%)	MM-150 (8.93%)							
25227334A>T	Y64N	/	/	3	MM-115 (54.2%)								
25227266A>T	N86K	/	/	/	MM-115 (44.27%)								
25245332G>T	A18D	/	COSM542	3	PCL-021 (43.8%)								
25227334A>C	Y64D	/	/	1	MM-410 (41.96%)								
25245351C>A	G12C	rs121913530	COSM516	4	MM-243 (40.5%)								
25227348G>C	A59G	/	COSM28518	3	MM-079 (23.13%)								
25227330C>A	S65I	/	/	/	PCL-009 (15.84%)								
25245350C>G	G12A	rs121913529	COSM522	3, 4	MM-140 (14.81%)								
25227342T>A	Q61L	rs121913240	COSM553	3, 4	MM-284 (9.54%)								
25245351C>T	G12S	rs121913530	COSM517	/	MM-004 (9.18%)								
25225657C>T	S136N	/	/	/	MM-202 (4.86%)								
25225627G>A	A146V	/	COSM19900	4	MM-445 (2.53%)								

*1: Chapman *et al.*, Nature 2011; 2: Walker *et al.*, Blood 2012; 3: Lohr *et al.*, Cancer Cell 2014; 4: Bolli *et al.*, Nature Communications 2014.

Supplementary Table 3B: Clinical and molecular characteristics of 49 MM/PCL patients carrying non-synonymous *KRAS* mutations

Sample name	*Disease*	*Variant (GRCh38)*	*% of mutated sequencing reads*	*AA change*	*Sex*	*Age*	*PP*†	*del(13)**	*del(17p)**	*1q gain**	*1p loss**	*t(4;14)*	*t(11;14)*	*t(14;16)*	*t(14;20)*	*HD*°
MM-004	MM	25245351C>T	9.18%	G12S	F	58	Gκ	-	na	na	na	-	-	+	-	na
MM-026	MM	25245321G>T/g.25245350C>T	32.94%/30.59%	Q22K/G12D	F	72	κ	-	-	-	na	-	+	-	-	na
MM-030	MM	25245350C>T	45.00%	G12D	M	69	Gλ	-	-	-	+	-	-	-	-	+
MM-038	MM	25227342T>C	40.82%	Q61R	F	67	κ	+	-	-	-	-	-	-	-	+
MM-039	MM	25245351C>G	46.94%	G12R	M	50	Gλ	-	-	-	na	-	-	-	-	+
MM-055	MM	25227341T>G	52.53%	Q61H	F	69	Gκ	-	-	-	na	-	+	-	-	-
MM-079	MM	25227348G>C	23.13%	A59G	F	74	Gκ+Gλ	-	-	-	-	-	-	-	-	+
MM-115	MM	25227334A>T/g.25227266A>T	54.20%/44.27%	Y64N/N86K	F	53	Gλ	+	-	na	na	-	+	-	-	-
MM-131	MM	25225628C>T	40.52%	A146T	M	73	Gκ	-	-	-	-	-	-	-	-	-
MM-140	MM	25245350C>G	14.81%	G12A	M	62	Gκ	-	-	-	na	-	+	-	-	-
MM-146	MM	25245350C>A	24.58%	G12V	M	68	Gλ	-	-	+	na	-	-	-	-	+
MM-149	MM	25245348C>A	29.13%	G13C	F	52	Gλ	-	-	-	-	-	-	-	-	+
MM-150	MM	25245350C>A	8.93%	G12V	F	68	Gλ	-	-	+	-	-	-	-	-	+
MM-151	MM	25227341T>G/g.25245347C>T	35.29%/9.35%	Q61H/G13D	F	71	Gλ	-	-	-	-	-	-	-	-	+
MM-152	MM	25245350C>T	31.69%	G12D	M	66	Gκ	-	-	-	na	-	-	-	-	+
MM-154	MM	25245350C>T	45.28%	G12D	F	71	Gκ	+	-	+	-	-	-	+	-	-
MM-159	MM	25245351C>G/g.25227342T>C	27.78%/3.75%	G12R/Q61R	M	56	κ	+	-	-	-	-	+	-	-	-
MM-200	MM	25227341T>G	23.74%	Q61H	F	63	Aλ	-	-	-	-	-	-	-	-	+
MM-202	MM	25227341T>G/g.25225657C>T	35.54%/4.86%	Q61H/S136N	M	64	Gκ	-	-	+	na	-	-	-	-	+
MM-219	MM	25245347C>T	5.88%	G13D	M	73	Aλ	+	-	-	-	-	-	-	-	+
MM-229	MM	25227341T>G	12.07%	Q61H	M	75	Gκ	-	-	-	-	-	-	-	-	-
MM-243	MM	25245351C>A	40.50%	G12C	F	68	Gκ	-	-	+	-	-	-	-	-	+
MM-269	MM	25225713T>G	3.07%	K117N	F	67	Gκ	+	-	-	-	-	-	-	-	+
MM-280	MM	25245321G>T	50.00%	Q22K	M	62	Gλ	-	-	-	-	-	+	-	-	-
MM-284	MM	25227342T>A	9.54%	Q61L	M	49	κ	na	-	-	-	-	+	-	-	-
MM-300	MM	25227341T>G	7.61%	Q61H	M	65	Gκ	-	-	na	na	-	-	-	-	na
MM-308	MM	25245347C>T	39.42%	G13D	M	58	Aκ	-	-	-	-	-	-	-	-	-
MM-310	MM	25225713T>G	47.09%	K117N	M	67	Gl	-	-	-	-	-	+	-	-	-
MM-327	MM	25227341T>G	37.34%	Q61H	F	62	absent	+	-	+	-	-	-	-	-	+
MM-335	MM	25245328C>G	18.60%	L19F	F	68	Aκ	+	-	-	-	-	-	+	-	-
MM-351	MM	25227342T>C	28.66%	Q61R	M	na	Aκ	-	-	-	-	-	-	-	-	-
MM-381	MM	25227341T>G	9.51%	Q61H	F	69	Gκ	-	-	-	-	-	-	-	na	na
MM-385	MM	25245347C>T	40.68%	G13D	F	76	Gλ	-	-	-	-	-	-	-	-	+
MM-386	MM	25245328C>G	42.73%	L19F	F	76	Aλ	-	-	na	na	-	na	na	na	na
MM-402	MM	25227341T>G	34.62%	Q61H	M	67	Gκ	+	-	-	-	-	+	-	-	-
MM-410	MM	25227334A>C	41.96%	Y64D	F	79	λ	-	-	+	-	-	-	-	-	-

Sample name	Disease	Variant (GRCh38)	% of mutated sequencing reads	AA change	Sex	Age	PP†	del(13)*	del(17p)*	1q gain*	1p loss*	t(4;14)	t(11;14)	t(14;16)	t(14;20)	HD°
MM-413	MM	25225628C>T	51.24%	A146T	F	51	Gκ	+	-	+	-	-	-	-	-	+
MM-429	MM	25245350C>T	21.47%	G12D	F	63	Gκ	-	-	+	-	-	-	-	-	+
MM-430	MM	25227341T>G	39.77%	Q61H	na	62	Gλ	-	-	-	-	-	-	-	-	-
MM-433	MM	25245350C>T	31.78%	G12D	M	72	κ	-	-	-	-	-	+	-	-	-
MM-440	MM	25227341T>G	38.00%	Q61H	na	61	Aκ	-	-	-	+	-	-	-	-	+
MM-441	MM	25227341T>G	8.45%	Q61H	na	na	Gλ	-	-	-	-	-	-	-	-	-
MM-442	sPCL	25227341T>G	45.93%	Q61H	F	65	Gκ	+	-	+	+	-	-	-	-	-
MM-445	MM	25245351C>G/g.25225627G>A	28.85%/2.53%	G12R/A146V	na	65	κ	+	-	+	-	-	+	-	-	-
PCL-001	pPCL	25245348C>A	48.64%	G13C	F	51	κ	+	-	na	na	-	-	-	+	na
PCL-009	sPCL	25227330C>A	15.84%	S65I	F	77	Gκ	+	-	+	-	+	-	-	-	-
PCL-021	pPCL	25245332G>T	43.80%	A18D	M	48	Gλ	+	-	+	-	+	-	-	-	-
PCL-038	pPCL	25245351C>G	42.42%	G12R	M	57	Gκ	+	-	+	+	-	-	-	-	+
PCL-046	pPCL	25227341T>G/g.25245350C>T	7.73%/9.01%	Q61H/G12D	F	50	κ	+	-	+	-	-	-	-	-	+

†Paraprotein; *del(13), del(17), 1p loss and 1q gain were determined by FISH. °HD = presence of the hyperdiploid status on the basis of FISH evaluation criteria.

Supplementary Table 3C: Clinical and molecular characteristics of the 167 MM/PCL patients analyzed for *KRAS* mutations.

Characteristic	*All patients (n=167)*		*KRAS wild type (n=118)*		*KRAS mutated (n=49)*		*P value*[a]
	N	*%*	*n*	*%*	*n*	*%*	
MM	132	*79*	89	*75.4*	43	*87.7*	
pPCL	24	*14.4*	20	*17*	4	*8.2*	n.s.
sPCL	11	*6.6*	9	*7.6*	2	*4.1*	
del(13q)	79	*47.6*	62	*52.5*	17	*35.4*	n.s.
chr 13 disomic patients	87	*52.4*	56	*47.5*	31	*64.6*	
del(17p)	18	*10.9*	18	*15.4*	0	*0*	0.0019
17p disomic patients	147	*89.1*	99	*84.6*	48	*100*	
1q gain	68	*43*	53	*46.5*	15	*34.1*	n.s.
1q disomic patients	90	*57*	61	*53.5*	29	*55.9*	
1p loss	18	*12.7*	14	*13.3*	4	*10.8*	n.s.
1p disomic patients	124	*87.3*	91	*86.7*	33	*89.2*	
IGH trx	82	*49.7*	64	*54.7*	18	*37.5*	n.s.
no *IGH* trx	83	*50.3*	53	*45.3*	30	*62.5*	
hyperdiploid	50	*32.9*	29	*26.6*	21	*48.8*	0.0124
non-hyperdiploid	102	*67.1*	80	*73.4*	22	*51.2*	

[a] Significance was assessed by Freeman-Halton extension of Fisher's exact test for disease type, and by Fisher's exact test for all other variables.

Supplementary Table 4A: Modulated genes between *BRAF*/*NRAS*/*KRAS* mutated and wild type patients

Gene	*SAM score*[‡]	*Gene*	*SAM score*
*PLEKHA1**	-5.163765	*ETV5**	6.24113
*ARL6IP5**	-4.342202	*CD300A**	5.77917
*ZHX1**	-4.311072	*SPRED2**	5.37656
*SMAD3**	-4.287826	*DUSP6**	4.36551
*AHNAK**	-4.268764	*NRXN1**	4.23105
*TBC1D1**	-4.237025	*MORC1**	4.20022
*MTMR4**	-4.167526	*F12**	4.15845
*COPZ2**	-4.103426	*RRBP1**	4.07138
*RASGRP1**	-4.07288	*TDO2**	4.03133
FLNB	-3.961337	*LGALS3BP*	3.97907
BMP2K	-3.918772	*PRKD2*	3.94335
RIOK1	-3.907891	*ACVR1B*	3.94093
TSHZ1	-3.904952	*ASPHD2*	3.92401
NOTCH2	-3.883492	*GPR56*	3.89699
ZNF681	-3.859508	*ACOXL*	3.85393
DHCR24	-3.82483	*SPRED1*	3.8092
LIFR	-3.819499	*LY9*	3.76526
ATL3	-3.818015	*ABI3BP*	3.74618
TGIF2	-3.794277	*DPEP1*	3.70693
GLO1	-3.776065	*TMEM184B*	3.70255
C6orf89	-3.76636	*PDE4D*	3.69971
ZNF570	-3.738105	*P2RY6*	3.68381
CYB5R1	-3.727057	*CASP4*	3.66862
PHTF2	-3.721193	*MIR221*	3.65834
DENND5A	-3.687141	*SEMA4D*	3.65632
CD52	-3.67123	*MAPKAPK2*	3.64609
ZADH2	-3.659897	*SIX4*	3.64407
RRP8	-3.635111		
BCL2	-3.627357		
JAZF1	-3.621626		
CD86	-3.612113		
LAMTOR3	-3.611917		
SAMSN1	-3.587501		
NTAN1	-3.586428		
PLCL1	-3.549426		
LRIG3	-3.547287		
PPM1L	-3.545335		
GRB2	-3.537068		
NECAP2	-3.522351		
KDM1B	-3.513606		
KAT6A	-3.51165		
BCL11A	-3.499956		
NEU1	-3.491002		
KIAA1147	-3.487048		
NUDT7	-3.455732		
SLC23A2	-3.446583		
LSS	-3.442877		
LYPD6B	-3.440096		
ARHGEF3	-3.436137		
CLN3	-3.423259		
INSIG1	-3.421565		
LYN	-3.382145		
APBB1IP	-3.378885		
MYL2	-3.372364		
TOP3A	-3.359096		
AKAP5	-3.358922		
RPSAP58	-3.357769		
EDN1	-3.355883		
SQLE	-3.353543		

*Differentially expressed gene at the highest stringency level. [‡]Negative scores indicate downregulated genes in mutated cases.

Supplementary Table 4B: Enriched biological processes in the MAPK mutation-associated 86 gene signature (*q* value<0.005)

ID	*Name*	*q value*	*Hit in Query List*
GO:0010563	negative regulation of phosphorus metabolic process	1.29E03	*SEMA4D, AKAP5, PDE4D, SPRED2, CD300A, DUSP6, SPRED1, SAMSN1, LYN, EDN1, SMAD3*
GO:0045936	negative regulation of phosphate metabolic process	1.29E03	*SEMA4D, AKAP5, PDE4D, SPRED2, CD300A, DUSP6, SPRED1, SAMSN1, LYN, EDN1, SMAD3*
GO:0042325	regulation of phosphorylation	1.29E03	*PRKD2, SEMA4D, AKAP5, PDE4D, SPRED2, MAPKAPK2, CD300A, ARL6IP5, DUSP6, GRB2, LAMTOR3, SPRED1, BCL2, PLCL1, ACVR1B, SAMSN1, LYN, EDN1, SMAD3*
GO:0001932	regulation of protein phosphorylation	1.29E03	*PRKD2, SEMA4D, AKAP5, PDE4D, SPRED2, MAPKAPK2, CD300A, DUSP6, LAMTOR3, SPRED1, BCL2, PLCL1, ACVR1B, SAMSN1, LYN, EDN1, SMAD3*
GO:0016126	sterol biosynthetic process	1.29E03	*DHCR24, INSIG1, SQLE, LSS, CYB5R1*
GO:1902531	regulation of intracellular signal transduction	1.29E03	*PRKD2, SEMA4D, RASGRP1, AKAP5, SPRED2, MAPKAPK2, TBC1D1, CD300A, ARL6IP5, DUSP6, GRB2, LAMTOR3, GPR56, PLEKHA1, SPRED1, BCL2, DENND5A, ARHGEF3, LYN, EDN1, NOTCH2*
GO:0023056	positive regulation of signaling	1.39E03	*PRKD2, SEMA4D, AKAP5, SPRED2, NRXN1, MAPKAPK2, CD300A, ARL6IP5, GRB2, LAMTOR3, GPR56, SPRED1, BCL2, ACVR1B, LYN, EDN1, NOTCH2, SMAD3*
GO:0010647	positive regulation of cell communication	1.39E03	*PRKD2, SEMA4D, AKAP5, SPRED2, NRXN1, MAPKAPK2, CD300A, ARL6IP5, GRB2, LAMTOR3, GPR56, SPRED1, BCL2, ACVR1B, LYN, EDN1, NOTCH2, SMAD3*
GO:1902533	positive regulation of intracellular signal transduction	1.39E03	*PRKD2, SEMA4D, AKAP5, SPRED2, MAPKAPK2, CD300A, ARL6IP5, LAMTOR3, GPR56, SPRED1, BCL2, LYN, EDN1, NOTCH2*
GO:0048584	positive regulation of response to stimulus	1.39E03	*PRKD2, SEMA4D, AKAP5, PDE4D, SPRED2, MAPKAPK2, CD86, CD300A, ARL6IP5, DUSP6, GRB2, LAMTOR3, GPR56, PLEKHA1, SPRED1, BCL2, ACVR1B, LYN, EDN1, NOTCH2, SMAD3*
GO:0001933	negative regulation of protein phosphorylation	1.39E03	*SEMA4D, PDE4D, SPRED2, CD300A, DUSP6, SPRED1, SAMSN1, LYN, SMAD3*
GO:0019220	regulation of phosphate metabolic process	1.63E03	*PRKD2, SEMA4D, RASGRP1, AKAP5, PDE4D, SPRED2, MAPKAPK2, TBC1D1, CD300A, ARL6IP5, DUSP6, GRB2, LAMTOR3, SPRED1, BCL2, PLCL1, DENND5A, ARHGEF3, ACVR1B, SAMSN1, LYN, EDN1, SMAD3*
GO:0002757	immune response activating signal transduction	1.63E03	*PRKD2, PDE4D, MAPKAPK2, CD86, CD300A, DUSP6, GRB2, PLEKHA1, BCL2, LYN*
GO:0051174	regulation of phosphorus metabolic process	1.63E03	*PRKD2, SEMA4D, RASGRP1, AKAP5, PDE4D, SPRED2, MAPKAPK2, TBC1D1, CD300A, ARL6IP5, DUSP6, GRB2, LAMTOR3, SPRED1, BCL2, PLCL1, DENND5A, ARHGEF3, ACVR1B, SAMSN1, LYN, EDN1, SMAD3*
GO:0009967	positive regulation of signal transduction	1.63E03	*PRKD2, SEMA4D, AKAP5, SPRED2, MAPKAPK2, CD300A, ARL6IP5, GRB2, LAMTOR3, GPR56, SPRED1, BCL2, ACVR1B, LYN, EDN1, NOTCH2, SMAD3*
GO:0002764	immune response regulating signaling pathway	2.03E03	*PRKD2, RASGRP1, PDE4D, MAPKAPK2, CD86, CD300A, DUSP6, GRB2, PLEKHA1, BCL2, LYN*
GO:0051094	positive regulation of developmental process	2.03E03	*PRKD2, SEMA4D, TGIF2, AKAP5, NRXN1, CD86, ETV5, BCL2, BCL11A, ACVR1B, SIX4, LYN, EDN1, NOTCH2, SMAD3*
GO:0023014	signal transduction by phosphorylation	2.03E03	*PRKD2, SPRED2, MAPKAPK2, CD300A, ARL6IP5, DUSP6, PPM1L, GRB2, LAMTOR3, SPRED1, ACVR1B, LYN, EDN1*
GO:0031324	negative regulation of cellular metabolic process	2.74E03	*SEMA4D, TGIF2, AKAP5, PDE4D, SPRED2, CLN3, CD300A, INSIG1, DUSP6, KAT6A, JAZF1, SPRED1, RRP8, BCL2, BCL11A, SAMSN1, LYN, ZHX1, EDN1, NOTCH2, SMAD3*
GO:0043407	negative regulation of MAP kinase activity	2.74E03	*SPRED2, CD300A, DUSP6, SPRED1, LYN*
GO:0042326	negative regulation of phosphorylation	2.74E03	*SEMA4D, PDE4D, SPRED2, CD300A, DUSP6, SPRED1, SAMSN1, LYN, SMAD3*
GO:0009892	negative regulation of metabolic process	2.74E03	*SEMA4D, TGIF2, AKAP5, PDE4D, SPRED2, CLN3, CD300A, INSIG1, DUSP6, KAT6A, JAZF1, SPRED1, RRP8, BCL2, BCL11A, ACVR1B, SAMSN1, LYN, ZHX1, EDN1, NOTCH2, SMAD3*
GO:0006468	protein phosphorylation	2.74E03	*PRKD2, SEMA4D, RIOK1, AKAP5, PDE4D, SPRED2, BMP2K, MAPKAPK2, CD300A, DUSP6, LAMTOR3, SPRED1, BCL2, PLCL1, ACVR1B, SAMSN1, LYN, EDN1, SMAD3*
GO:2000026	regulation of multicellular organismal development	2.76E03	*PRKD2, SEMA4D, TGIF2, NEU1, AKAP5, NRXN1, BMP2K, CD86, DUSP6, ETV5, GPR56, BCL2, BCL11A, ACVR1B, SIX4, LYN, EDN1, NOTCH2, SMAD3*

ID	*Name*	*q value*	*Hit in Query List*
GO:0002253	activation of immune response	2.77E03	*PRKD2, PDE4D, MAPKAPK2, CD86, CD300A, DUSP6, GRB2, PLEKHA1, BCL2, LYN*
GO:0050853	B cell receptor signaling pathway	3.23E03	*CD300A, PLEKHA1, BCL2, LYN*
GO:0002768	immune response regulating cell surface receptor signaling pathway	3.29E03	*PRKD2, RASGRP1, PDE4D, CD86, CD300A, GRB2, PLEKHA1, BCL2, LYN*
GO:0030334	regulation of cell migration	3.29E03	*DPEP1, PRKD2, SEMA4D, P2RY6, CD300A, GPR56, BCL2, ACVR1B, LYN, EDN1, SMAD3*
GO:0051270	regulation of cellular component movement	3.29E03	*DPEP1, PRKD2, SEMA4D, PDE4D, P2RY6, CD300A, GPR56, BCL2, ACVR1B, LYN, EDN1, SMAD3*
GO:0000165	MAPK cascade	3.29E03	*PRKD2, SPRED2, MAPKAPK2, CD300A, ARL6IP5, DUSP6, PPM1L, GRB2, LAMTOR3, SPRED1, LYN, EDN1*
GO:0032269	negative regulation of cellular protein metabolic process	3.62E03	*SEMA4D, PDE4D, SPRED2, CLN3, CD300A, DUSP6, SPRED1, SAMSN1, LYN, EDN1, SMAD3*
GO:0016310	phosphorylation	3.73E03	*PRKD2, SEMA4D, RIOK1, AKAP5, PDE4D, SPRED2, BMP2K, MAPKAPK2, CD300A, ARL6IP5, DUSP6, PPM1L, GRB2, LAMTOR3, SPRED1, BCL2, PLCL1, ACVR1B, SAMSN1, LYN, EDN1, SMAD3*
GO:0006695	cholesterol biosynthetic process	3.73E03	*DHCR24, INSIG1, SQLE, LSS*
GO:0007167	enzyme linked receptor protein signaling pathway	4.06E03	*LIFR, PRKD2, TGIF2, MTMR4, MAPKAPK2, CD86, DUSP6, PPM1L, GRB2, PLEKHA1, ARHGEF3, ACVR1B, LYN, NOTCH2, SMAD3*
GO:0043583	ear development	4.06E03	*LRIG3, MAPKAPK2, INSIG1, TSHZ1, BCL2, SIX4, EDN1*
GO:0050851	antigen receptor mediated signaling pathway	4.06E03	*PRKD2, PDE4D, CD300A, PLEKHA1, BCL2, LYN*
GO:0031399	regulation of protein modification process	4.06E03	*PRKD2, SEMA4D, AKAP5, PDE4D, SPRED2, MAPKAPK2, CD300A, DUSP6, LAMTOR3, SPRED1, BCL2, PLCL1, ACVR1B, SAMSN1, LYN, EDN1, SMAD3*
GO:0043408	regulation of MAPK cascade	4.06E03	*PRKD2, SPRED2, MAPKAPK2, CD300A, ARL6IP5, DUSP6, GRB2, LAMTOR3, SPRED1, LYN, EDN1*
GO:2000145	regulation of cell motility	4.08E03	*DPEP1, PRKD2, SEMA4D, P2RY6, CD300A, GPR56, BCL2, ACVR1B, LYN, EDN1, SMAD3*
GO:0050776	regulation of immune response	4.11E03	*PRKD2, RASGRP1, PDE4D, MAPKAPK2, CD86, CD300A, DUSP6, GRB2, PLEKHA1, BCL2, SAMSN1, LYN, SMAD3*
GO:0018193	peptidyl amino acid modification	4.90E03	*PRKD2, SEMA4D, PDE4D, SPRED2, MAPKAPK2, CD300A, KAT6A, SPRED1, ASPHD2, BCL2, PLCL1, ACVR1B, SAMSN1, LYN*

Supplementary Table 5A: Description of the 150 genes at the top of the ranked list in GSEA analysis of U266 treated *versus* control cells

GENE_SYMBOL	*GENE_TITLE*	*SCORE*
INHBE	inhibin, beta E	0.676317
CTH	cystathionase (cystathionine gamma-lyase)	0.634
STC2	stanniocalcin 2	0.600332
FBXW10	F-box and WD-40 domain protein 10	0.574939
TCP11L2	t-complex 11 (mouse) like 2	0.532132
ESRP1	epithelial splicing regulatory protein 1	0.509604
DDIT4	DNA-damage-inducible transcript 4	0.498076
TRIB3	tribbles homolog 3 (Drosophila)	0.474272
GRB10	growth factor receptor-bound protein 10	0.471464
ASS1	argininosuccinate synthetase 1	0.458725
TMEM154	transmembrane protein 154	0.457523
GDF15	growth differentiation factor 15	0.452052
FAM106CP	family with sequence similarity 106, member C, pseudogene	0.448272
SESN2	sestrin 2	0.437719
BEX2	brain expressed X-linked 2	0.422433
PCK2	phosphoenolpyruvate carboxykinase 2 (mitochondrial)	0.41365
SLC35F1	solute carrier family 35, member F1	0.405951
CREB5	cAMP responsive element binding protein 5	0.403822
SLC10A5	solute carrier family 10 (sodium/bile acid cotransporter family), member 5	0.401765
HMOX1	heme oxygenase (decycling) 1	0.39905
SNORD116-18	small nucleolar RNA, C/D box 116-18	0.397014
FDPSP2	farnesyl diphosphate synthase pseudogene 2	0.385425
VSNL1	visinin-like 1	0.381415
SNORD116-25	small nucleolar RNA, C/D box 116-25	0.38046
SLC7A11	solute carrier family 7, (cationic amino acid transporter, y+ system) member 11	0.379498
LOC101928082	uncharacterized LOC101928082	0.371708
EIF4EBP1	eukaryotic translation initiation factor 4E binding protein 1	0.36092
TUBE1	tubulin, epsilon 1	0.358196
TRAV27	T cell receptor alpha variable 27	0.356685
CBS	cystathionine-beta-synthase	0.354561
ISY1	ISY1 splicing factor homolog (S. cerevisiae)	0.354496
SNORD115-24	small nucleolar RNA, C/D box 115-24	0.353983
ANK2	ankyrin 2, neuronal	0.351251
LOC101928574	uncharacterized LOC101928574	0.351248
VLDLR	very low density lipoprotein receptor	0.34859
SLC43A1	solute carrier family 43, member 1	0.342847
CXorf61	chromosome X open reading frame 61	0.342714
STON1	stonin 1	0.341989
CTAGE5	CTAGE family, member 5	0.341841
PRSS8	protease, serine, 8 (prostasin)	0.340513
TRAV10	T cell receptor alpha variable 10	0.340494
DDIT3	DNA-damage-inducible transcript 3	0.336389
RPS10P7	ribosomal protein S10 pseudogene 7	0.334938
CHAC1	ChaC, cation transport regulator homolog 1 (E. coli)	0.332567
FAM227B	family with sequence similarity 227, member B	0.332222
ALDH2	aldehyde dehydrogenase 2 family (mitochondrial)	0.331961
SLC6A9	solute carrier family 6 (neurotransmitter transporter, glycine), member 9	0.330724
MIR186	microRNA 186	0.328755
ZNF763	zinc finger protein 763	0.328666
HIST1H2AJ	histone cluster 1, H2aj	0.327899
RNU5D-1	RNA, U5D small nuclear 1	0.323913
ALOX5AP	arachidonate 5-lipoxygenase-activating protein	0.32156
FBXO32	F-box protein 32	0.32125
ZC3H6	zinc finger CCCH-type containing 6	0.316641
GPT2	glutamic pyruvate transaminase (alanine aminotransferase) 2	0.314711

GENE_SYMBOL	GENE_TITLE	SCORE
COLGALT2	collagen beta(1-O)galactosyltransferase 2	0.314565
WEE2-AS1	WEE2 antisense RNA 1	0.314304
PPM1M	protein phosphatase 1M (PP2C domain containing)	0.313921
SNORA31	small nucleolar RNA, H/ACA box 31	0.313363
C11orf74	chromosome 11 open reading frame 74	0.313307
ZMAT1	zinc finger, matrin type 1	0.313217
RND3	Rho family GTPase 3	0.309211
CLGN	calmegin	0.305442
UGT1A10	UDP glucuronosyltransferase 1 family, polypeptide A10	0.30265
CIR1	corepressor interacting with RBPJ	0.301674
CXorf22	chromosome X open reading frame 22	0.299363
ALDH1L2	aldehyde dehydrogenase 1 family, member L2	0.298943
RBM11	RNA binding motif protein 11	0.298281
SNORD116-13	small nucleolar RNA, C/D box 116-13	0.298082
TTLL1	tubulin tyrosine ligase-like family, member 1	0.297329
CEBPB	CCAAT/enhancer binding protein (C/EBP), beta	0.296077
C2orf27A	chromosome 2 open reading frame 27A	0.295393
FAM129A	family with sequence similarity 129, member A	0.286598
KIAA1257	KIAA1257	0.285531
LOC644717	sarcoma antigen 2, pseudogene	0.285277
AGMO	alkylglycerol monooxygenase	0.280998
PSAT1	phosphoserine aminotransferase 1	0.278228
THAP9-AS1	THAP9 antisense RNA 1	0.277759
SMPX	small muscle protein, X-linked	0.277239
COBLL1	COBL-like 1	0.272505
WNT5A	wingless-type MMTV integration site family, member 5A	0.271617
MIR29A	microRNA 29a	0.271519
CBX4	chromobox homolog 4 (Pc class homolog, Drosophila)	0.270723
MT2A	metallothionein 2A	0.267114
ATF3	activating transcription factor 3	0.26646
PDLIM3	PDZ and LIM domain 3	0.265137
LYPLA1	lysophospholipase I	0.263954
PRH2	proline-rich protein HaeIII subfamily 2	0.2622
SMIM14	small integral membrane protein 14	0.261547
SCN4A	sodium channel, voltage-gated, type IV, alpha	0.260615
PEX5L	peroxisomal biogenesis factor 5-like	0.260427
RAB39B	RAB39B, member RAS oncogene family	0.259919
BEST1	bestrophin 1	0.259136
MIR2682	microRNA 2682	0.259071
LOC339803	hypothetical protein LOC339803	0.25466
MAP1LC3B2	microtubule-associated protein 1 light chain 3 beta 2	0.254636
FAAH2	fatty acid amide hydrolase 2	0.254589
RNU5B-1	RNA, U5B small nuclear 1	0.25144
ZNF117	zinc finger protein 117	0.251039
SCARNA9	small Cajal body-specific RNA 9	0.250541
NR5A2	nuclear receptor subfamily 5, group A, member 2	0.249632
CHDC2	calponin homology domain containing 2	0.249293
CCPG1	cell cycle progression 1	0.248701
ZNF674	zinc finger protein 674	0.248269
PIP5KL1	phosphatidylinositol-4-phosphate 5-kinase-like 1	0.248112
INPP1	inositol polyphosphate-1-phosphatase	0.247267
IFRD1	interferon-related developmental regulator 1	0.245181
LYPLAL1	lysophospholipase-like 1	0.245028
KIF21B	kinesin family member 21B	0.244496
LRRC37A3	leucine rich repeat containing 37, member A3	0.244306
TBC1D3P2	TBC1 domain family, member 3 pseudogene 2	0.243629
OPN1LW	opsin 1 (cone pigments), long-wave-sensitive (color blindness, protan)	0.243267

GENE_SYMBOL	GENE_TITLE	SCORE
PCDHA1	protocadherin alpha 1	0.243149
IGHV1-45	immunoglobulin heavy variable 1-45	0.241021
ANKRD31	ankyrin repeat domain 31	0.240202
KIF27	kinesin family member 27	0.239263
OR2M3	olfactory receptor, family 2, subfamily M, member 3	0.238097
MT1X	metallothionein 1X	0.237609
COX7B	cytochrome c oxidase subunit VIIb	0.23747
ZNF429	zinc finger protein 429	0.237151
MBNL2	muscleblind-like 2 (Drosophila)	0.235401
ACSM3	acyl-CoA synthetase medium-chain family member 3	0.2354
CYP19A1	cytochrome P450, family 19, subfamily A, polypeptide 1	0.234959
SLC1A4	solute carrier family 1 (glutamate/neutral amino acid transporter), member 4	0.233497
TP53	tumor protein p53 (Li-Fraumeni syndrome)	0.233082
RGMB	RGM domain family, member B	0.229311
SNORD116-6	small nucleolar RNA, C/D box 116-6	0.226827
ARHGAP10	Rho GTPase activating protein 10	0.226407
MST1	macrophage stimulating 1 (hepatocyte growth factor-like)	0.226056
RFPL4AL1	ret finger protein-like 4A-like 1	0.225059
FAM45B	family with sequence similarity 45, member B	0.224498
ERO1LB	ERO1-like beta (S. cerevisiae)	0.223822
FBXO48	F-box protein 48	0.223482
C5orf28	chromosome 5 open reading frame 28	0.22115
DDR2	discoidin domain receptor family, member 2	0.22074
TNFRSF10B	tumor necrosis factor receptor superfamily, member 10b	0.220335
AMY2B	amylase, alpha 2B (pancreatic)	0.219811
SNORD116-26	small nucleolar RNA, C/D box 116-26	0.217503
TRNASUP1	transfer RNA suppressor 1 (anticodon UUA)	0.217355
PBLD	phenazine biosynthesis-like protein domain containing	0.217007
IGHV1-69	immunoglobulin heavy variable 1-69	0.215382
IRAK1BP1	interleukin-1 receptor-associated kinase 1 binding protein 1	0.214788
ARHGAP9	Rho GTPase activating protein 9	0.214722
PHGDH	phosphoglycerate dehydrogenase	0.213742
PYCR1	pyrroline-5-carboxylate reductase 1	0.213522
SCARNA9L	small Cajal body-specific RNA 9-like	0.213237
DEPTOR	DEP domain containing MTOR-interacting protein	0.212935
BCAT1	branched chain aminotransferase 1, cytosolic	0.212422
SHMT2	serine hydroxymethyltransferase 2 (mitochondrial)	0.211524
ALPK2	alpha-kinase 2	0.211106

Supplementary Table 5B: Description of the 150 genes at the bottom of the ranked list in GSEA analysis of U266 treated *versus* control cells

GENE_SYMBOL	*GENE_TITLE*	*SCORE*
OR4M1	olfactory receptor, family 4, subfamily M, member 1	-0.18006
PRLR	prolactin receptor	-0.18045
FAM19A1	family with sequence similarity 19 (chemokine (C-C motif)-like), member A1	-0.18057
TNFSF13	tumor necrosis factor (ligand) superfamily, member 13	-0.18059
TUBA3E	tubulin, alpha 3e	-0.18091
FOS	v-fos FBJ murine osteosarcoma viral oncogene homolog	-0.181
MIR146A	microRNA 146a	-0.18117
EPGN	epithelial mitogen homolog (mouse)	-0.18137
SNORD27	small nucleolar RNA, C/D box 27	-0.18153
LOC339524	pothetical LOC339524	-0.18153
OR7D4	olfactory receptor, family 7, subfamily D, member 4	-0.18161
IL5RA	interleukin 5 receptor, alpha	-0.18194
SERPINB3	serpin peptidase inhibitor, clade B (ovalbumin), member 3	-0.18204
OR7E5P	olfactory receptor, family 7, subfamily E, member 5 pseudogene	-0.1823
WBP4	WW domain binding protein 4 (formin binding protein 21)	-0.18311
STARD4	START domain containing 4, sterol regulated	-0.18332
VAV3	vav 3 oncogene	-0.18398
S100A10	S100 calcium binding protein A10	-0.18437
SNORD115-32	small nucleolar RNA, C/D box 115-32	-0.18475
MIR101-2	microRNA 101-2	-0.1848
C4BPB	complement component 4 binding protein, beta	-0.18487
CNN2	calponin 2	-0.18517
OR2J3	olfactory receptor, family 2, subfamily J, member 3	-0.18566
MPV17L	MPV17 mitochondrial membrane protein-like	-0.18629
SMPDL3A	sphingomyelin phosphodiesterase, acid-like 3A	-0.18643
ZNF492	zinc finger protein 492	-0.18661
IGHV3-35	immunoglobulin heavy variable 3-35	-0.18676
OR3A1	olfactory receptor, family 3, subfamily A, member 1	-0.18715
CYP2B7P1	cytochrome P450, family 2, subfamily B, polypeptide 7 pseudogene 1	-0.18722
SQLE	squalene epoxidase	-0.1877
CYP4A22	cytochrome P450, family 4, subfamily A, polypeptide 22	-0.18797
PDIA3	protein disulfide isomerase family A, member 3	-0.18818
OR13J1	olfactory receptor, family 13, subfamily J, member 1	-0.18828
SAA2	serum amyloid A2	-0.18866
OR56A3	olfactory receptor, family 56, subfamily A, member 3	-0.18884
GAS6	growth arrest-specific 6	-0.18925
PLN	phospholamban	-0.18931
C1QTNF9B-AS1	C1QTNF9B antisense RNA 1	-0.18973
LAX1	lymphocyte transmembrane adaptor 1	-0.19029
EGFL6	EGF-like-domain, multiple 6	-0.19069
RBM46	RNA binding motif protein 46	-0.19214
RFPL1	ret finger protein-like 1	-0.19246
SLCO4A1	solute carrier organic anion transporter family, member 4A1	-0.19335
BTN2A3P	butyrophilin, subfamily 2, member A3, pseudogene	-0.19382
EFCAB2	EF-hand calcium binding domain 2	-0.19434
GYPB	glycophorin B (MNS blood group)	-0.19494
KRTAP19-8	keratin associated protein 19-8	-0.19593
PBK	PDZ binding kinase	-0.19827
IGHV3-33	immunoglobulin heavy variable 3-33	-0.19842
HSPH1	heat shock 105kDa/110kDa protein 1	-0.19858
MIR377	microRNA 377	-0.19874
PRICKLE1	prickle homolog 1 (Drosophila)	-0.19891
ZNF300P1	zinc finger protein 300 pseudogene 1 (functional)	-0.1992
CDC25A	cell division cycle 25A	-0.20011
POU2F2	POU domain, class 2, transcription factor 2	-0.20045

GENE_SYMBOL	GENE_TITLE	SCORE
SHCBP1	SHC SH2-domain binding protein 1	-0.20052
TRAV8-3	T cell receptor alpha variable 8-3	-0.20052
PSG10P	pregnancy specific beta-1-glycoprotein 10, pseudogene	-0.2013
BCL3	B-cell CLL/lymphoma 3	-0.20196
RPS27A	ribosomal protein S27a	-0.20228
SNORA2A	small nucleolar RNA, H/ACA box 2A	-0.20261
OR1N1	olfactory receptor, family 1, subfamily N, member 1	-0.20264
OR10G3	olfactory receptor, family 10, subfamily G, member 3	-0.20269
PKP2	plakophilin 2	-0.20317
RNU6-76P	RNA, U6 small nuclear 76, pseudogene	-0.20332
PGM2L1	phosphoglucomutase 2-like 1	-0.20358
DTX3L	deltex 3-like (Drosophila)	-0.20393
IFIT1	interferon-induced protein with tetratricopeptide repeats 1	-0.20488
GOLGA8DP	golgin A8 family, member D, pseudogene	-0.20604
NETO1	neuropilin (NRP) and tolloid (TLL)-like 1	-0.20608
ADAM21	ADAM metallopeptidase domain 21	-0.2074
STON1-GTF2A1L	STON1-GTF2A1L readthrough	-0.2077
PTP4A3	protein tyrosine phosphatase type IVA, member 3	-0.20809
MIR17HG	microRNA host gene 1 (non-protein coding)	-0.20824
PCDH11Y	protocadherin 11 Y-linked	-0.2088
FAM172BP	family with sequence similarity 172, member B, pseudogene	-0.21178
HMGCR	3-hydroxy-3-methylglutaryl-Coenzyme A reductase	-0.212
MIR224	microRNA 224	-0.2121
LOC101929115	uncharacterized LOC101929115	-0.21248
TMEM75	transmembrane protein 75	-0.21334
PPT2-EGFL8	PPT2-EGFL8 readthrough (NMD candidate)	-0.21366
SPRED2	sprouty-related, EVH1 domain containing 2	-0.21369
KLRC2	killer cell lectin-like receptor subfamily C, member 2	-0.21392
MIR103A2	microRNA 103a2	-0.21466
LOC730102	hypothetical protein LOC730102	-0.21508
TLR7	toll-like receptor 7	-0.21603
FASN	fatty acid synthase	-0.21608
MSMO1	methylsterol monooxygenase 1	-0.21786
OR2T29	olfactory receptor, family 2, subfamily T, member 29	-0.2184
TMPRSS11B	transmembrane protease, serine 11B	-0.22077
RPL13A	ribosomal protein L13a	-0.22087
EGR1	early growth response 1	-0.22169
C3orf14	chromosome 3 open reading frame 14	-0.22297
SLA	Src-like-adaptor	-0.2233
UGP2	UDP-glucose pyrophosphorylase 2	-0.22551
BCL6	B-cell CLL/lymphoma 6 (zinc finger protein 51)	-0.22575
IGKV4-1	immunoglobulin kappa variable 4-1	-0.22598
E2F2	E2F transcription factor 2	-0.23065
LOC100288637	OTU deubiquitinase 7A pseudogene	-0.23075
KRTAP21-1	keratin associated protein 21-1	-0.23221
SPANXN1	SPANX family, member N1	-0.23226
UGT2B28	UDP glucuronosyltransferase 2 family, polypeptide B28	-0.23258
KRTAP5-4	keratin associated protein 5-4	-0.23337
ACOXL	acyl-Coenzyme A oxidase-like	-0.23394
PLA2G10	phospholipase A2, group X	-0.23428
LDLR	low density lipoprotein receptor (familial hypercholesterolemia)	-0.23852
C9orf153	chromosome 9 open reading frame 153	-0.24111
TPRX1	tetra-peptide repeat homeobox 1	-0.24176
NAIP	NLR family, apoptosis inhibitory protein	-0.24236
AKAP5	A kinase (PRKA) anchor protein 5	-0.24382
HMGCS1	3-hydroxy-3-methylglutaryl-Coenzyme A synthase 1 (soluble)	-0.25189
MYH8	myosin, heavy chain 8, skeletal muscle, perinatal	-0.2531

GENE_SYMBOL	GENE_TITLE	SCORE
PSG1	pregnancy specific beta-1-glycoprotein 1	-0.25377
ZNF718	zinc finger protein 718	-0.26188
MROH7	maestro heat-like repeat family member 7	-0.26352
LBH	limb bud and heart development homolog (mouse)	-0.26527
ID3	inhibitor of DNA binding 3, dominant negative helix-loop-helix protein	-0.26606
PRDM9	PR domain containing 9	-0.2697
DUSP6	dual specificity phosphatase 6	-0.27505
ZFP36	zinc finger protein 36, C3H type, homolog (mouse)	-0.27713
IL6	interleukin 6 (interferon, beta 2)	-0.28043
RNU6-23P	RNA, U6 small nuclear 23, pseudogene	-0.28877
TBC1D3B	TBC1 domain family, member 3B	-0.29116
ETV4	ets variant gene 4 (E1A enhancer binding protein, E1AF)	-0.29659
MYOT	myotilin	-0.29938
OCM2	oncomodulin 2	-0.31006
IDI1	isopentenyl-diphosphate delta isomerase 1	-0.31097
SPRED1	sprouty-related, EVH1 domain containing 1	-0.31603
IFNA7	interferon, alpha 7	-0.31626
MIR142	microRNA 142	-0.31715
DHCR24	24-dehydrocholesterol reductase	-0.31717
PRR23A	proline rich 23A	-0.33145
TDGF1	teratocarcinoma-derived growth factor 1	-0.33705
INSIG1	insulin induced gene 1	-0.34427
GAGE1	G antigen 1	-0.34578
LOC286359	hypothetical LOC286359	-0.35466
ETV5	ets variant gene 5 (ets-related molecule)	-0.38229
IFNA10	interferon, alpha 10	-0.38231
ARL2-SNX15	ARL2-SNX15 readthrough (NMD candidate)	-0.38978
PARP9	poly (ADP-ribose) polymerase family, member 9	-0.39203
ZNF578	zinc finger protein 578	-0.40315
TRIM51	tripartite motif-containing 51	-0.40741
CCR1	chemokine (C-C motif) receptor 1	-0.40874
TRAV5	T cell receptor alpha variable 5	-0.41448
OR13C2	olfactory receptor, family 13, subfamily C, member 2	-0.42899
CD180	CD180 molecule	-0.43024
MIR148A	microRNA 148a	-0.43208
TRAV17	T cell receptor alpha variable 17	-0.46508
VTRNA1-1	vault RNA 1-1	-0.47056
GPRC5D	G protein-coupled receptor, family C, group 5, member D	-0.5071

Supplementary Table 6A: Gene sets enriched in U266 control cells

NAME	*NES*	*FDR q-value*
ROSTY_CERVICAL_CANCER_PROLIFERATION_CLUSTER	-2.50567	0
KOBAYASHI_EGFR_SIGNALING_24HR_DN	-2.47026	0
DUTERTRE_ESTRADIOL_RESPONSE_24HR_UP	-2.45821	0
CROONQUIST_NRAS_SIGNALING_DN	-2.43549	0
CROONQUIST_IL6_DEPRIVATION_DN	-2.42036	0
GRAHAM_NORMAL_QUIESCENT_VS_NORMAL_DIVIDING_DN	-2.41937	0
ZHANG_TLX_TARGETS_UP	-2.41358	0
LEE_EARLY_T_LYMPHOCYTE_UP	-2.39146	0
ZHAN_MULTIPLE_MYELOMA_PR_UP	-2.37026	0
CHANG_CYCLING_GENES	-2.3691	0
SCHMIDT_POR_TARGETS_IN_LIMB_BUD_UP	-2.36034	0
SOTIRIOU_BREAST_CANCER_GRADE_1_VS_3_UP	-2.35916	0
KANG_DOXORUBICIN_RESISTANCE_UP	-2.35159	0
KONG_E2F3_TARGETS	-2.23723	4.46E-05
GOBERT_OLIGODENDROCYTE_DIFFERENTIATION_UP	-2.24137	4.67E-05
MANALO_HYPOXIA_DN	-2.24976	4.89E-05
BENPORATH_ES_2	-2.25888	5.13E-05
REACTOME_CHOLESTEROL_BIOSYNTHESIS	-2.26001	5.40E-05
GSE15750_DAY6_VS_DAY10_EFF_CD8_TCELL_UP	-2.26123	5.70E-05
ISHIDA_E2F_TARGETS	-2.26645	6.04E-05
FURUKAWA_DUSP6_TARGETS_PCI35_DN	-2.31819	6.41E-05
VERNELL_RETINOBLASTOMA_PATHWAY_UP	-2.33317	6.84E-05
ZHOU_CELL_CYCLE_GENES_IN_IR_RESPONSE_24HR	-2.33854	7.33E-05
WINNEPENNINCKX_MELANOMA_METASTASIS_UP	-2.22891	8.12E-05
BLUM_RESPONSE_TO_SALIRASIB_DN	-2.23351	8.46E-05
ZHOU_CELL_CYCLE_GENES_IN_IR_RESPONSE_6HR	-2.20068	9.85E-05
HORTON_SREBF_TARGETS	-2.2023	1.02E-04
GOLDRATH_EFF_VS_MEMORY_CD8_TCELL_UP	-2.20359	1.05E-04
ODONNELL_TFRC_TARGETS_DN	-2.21489	1.09E-04
SARRIO_EPITHELIAL_MESENCHYMAL_TRANSITION_UP	-2.21638	1.13E-04
ZHANG_TLX_TARGETS_60HR_DN	-2.22135	1.17E-04
ODONNELL_TARGETS_OF_MYC_AND_TFRC_DN	-2.18752	1.20E-04
WANG_RESPONSE_TO_GSK3_INHIBITOR_SB216763_DN	-2.18812	1.23E-04
GRAHAM_CML_DIVIDING_VS_NORMAL_QUIESCENT_UP	-2.18997	1.27E-04
MITSIADES_RESPONSE_TO_APLIDIN_DN	-2.17531	1.75E-04
CHIANG_LIVER_CANCER_SUBCLASS_PROLIFERATION_UP	-2.16301	2.27E-04
KEGG_STEROID_BIOSYNTHESIS	-2.12567	2.63E-04
HOFFMANN_LARGE_TO_SMALL_PRE_BII_LYMPHOCYTE_UP	-2.12669	2.69E-04
BURTON_ADIPOGENESIS_3	-2.14326	2.77E-04
AMUNDSON_GAMMA_RADIATION_RESPONSE	-2.11155	3.49E-04
GSE15750_DAY6_VS_DAY10_TRAF6KO_EFF_CD8_TCELL_UP	-2.11248	3.58E-04
PODAR_RESPONSE_TO_ADAPHOSTIN_DN	-2.10595	4.15E-04
ZHANG_TLX_TARGETS_36HR_DN	-2.10456	4.29E-04
CAFFAREL_RESPONSE_TO_THC_DN	-2.08356	9.54E-04
BENPORATH_PROLIFERATION	-2.08173	9.56E-04
FUJII_YBX1_TARGETS_DN	-2.07129	0.001047
M_PHASE_OF_MITOTIC_CELL_CYCLE	-2.06083	0.001171
GSE30962_PRIMARY_VS_SECONDARY_ACUTE_LCMV_INF_CD8_TCELL_UP	-2.06335	0.001177

NAME	*NES*	*FDR q-value*
LY_AGING_OLD_DN	-2.05885	0.001186
KEGG_TERPENOID_BACKBONE_BIOSYNTHESIS	-2.05962	0.001189
YU_MYC_TARGETS_UP	-2.06141	0.001195
TIEN_INTESTINE_PROBIOTICS_24HR_UP	-2.05638	0.001202
MARKEY_RB1_CHRONIC_LOF_UP	-2.0486	0.001329
MITOSIS	-2.04949	0.001335
MARKEY_RB1_ACUTE_LOF_DN	-2.0437	0.001337
EGUCHI_CELL_CYCLE_RB1_TARGETS	-2.0454	0.001342
REACTOME_DNA_REPLICATION	-2.04318	0.001349
PENG_LEUCINE_DEPRIVATION_DN	-2.04199	0.001396
GARGALOVIC_RESPONSE_TO_OXIDIZED_PHOSPHOLIPIDS_TURQUOISE_DN	-2.03984	0.001425
LY_AGING_PREMATURE_DN	-2.03722	0.001453
M_PHASE	-2.0264	0.001719
KAMMINGA_EZH2_TARGETS	-2.02676	0.001731
PENG_GLUTAMINE_DEPRIVATION_DN	-2.0262	0.00174
FINETTI_BREAST_CANCER_KINOME_RED	-2.01831	0.001986
REACTOME_G1_S_SPECIFIC_TRANSCRIPTION	-2.01672	0.002018
GARY_CD5_TARGETS_DN	-2.00731	0.002346
CROONQUIST_NRAS_VS_STROMAL_STIMULATION_DN	-2.00023	0.002594
PID_FOXM1PATHWAY	-2.00028	0.002632
WILCOX_PRESPONSE_TO_ROGESTERONE_UP	-1.99054	0.00315
LIU_IL13_PRIMING_MODEL	-1.98765	0.00334
SMIRNOV_RESPONSE_TO_IR_6HR_DN	-1.98641	0.003409
MISSIAGLIA_REGULATED_BY_METHYLATION_DN	-1.98407	0.003505
MOLENAAR_TARGETS_OF_CCND1_AND_CDK4_DN	-1.98123	0.003668
HORIUCHI_WTAP_TARGETS_DN	-1.9723	0.0042
REACTOME_MITOTIC_M_M_G1_PHASES	-1.97297	0.004201
WHITEFORD_PEDIATRIC_CANCER_MARKERS	-1.9688	0.004428
REGULATION_OF_MITOSIS	-1.96696	0.004504
LI_WILMS_TUMOR_ANAPLASTIC_UP	-1.96585	0.004538
INACTIVATION_OF_MAPK_ACTIVITY	-1.96465	0.004572
AFFAR_YY1_TARGETS_DN	-1.96296	0.004656
GSE10239_NAIVE_VS_DAY4.5_EFF_CD8_TCELL_DN	-1.95866	0.004966
IKEDA_MIR133_TARGETS_UP	-1.9544	0.005281
MORI_LARGE_PRE_BII_LYMPHOCYTE_UP	-1.95038	0.005527
REACTOME_CELL_CYCLE_MITOTIC	-1.94704	0.005724
PUJANA_XPRSS_INT_NETWORK	-1.94754	0.005756
SHEDDEN_LUNG_CANCER_POOR_SURVIVAL_A6	-1.94109	0.006255
REACTOME_MITOTIC_PROMETAPHASE	-1.93893	0.006489
MYELOID_LEUKOCYTE_DIFFERENTIATION	-1.93734	0.006574
PUJANA_BRCA_CENTERED_NETWORK	-1.93737	0.006637
FOURNIER_ACINAR_DEVELOPMENT_LATE_2	-1.9316	0.007106
MUELLER_PLURINET	-1.92509	0.007672
KAUFFMANN_MELANOMA_RELAPSE_UP	-1.92513	0.007756
REICHERT_MITOSIS_LIN9_TARGETS	-1.92548	0.007796
PID_PLK1_PATHWAY	-1.91885	0.008409
TARTE_PLASMA_CELL_VS_PLASMABLAST_DN	-1.91183	0.009402
GSE24634_TEFF_VS_TCONV_DAY7_IN_CULTURE_UP	-1.90901	0.009785
VECCHI_GASTRIC_CANCER_EARLY_UP	-1.90755	0.009959

NAME	*NES*	*FDR q-value*
AIYAR_COBRA1_TARGETS_DN	-1.90127	0.010795
CHIARADONNA_NEOPLASTIC_TRANSFORMATION_KRAS_UP	-1.90134	0.010894
REACTOME_GLUCURONIDATION	-1.89377	0.011913
WHITFIELD_CELL_CYCLE_LITERATURE	-1.89411	0.011991
FERREIRA_EWINGS_SARCOMA_UNSTABLE_VS_STABLE_UP	-1.88504	0.013656
CHROMOSOME	-1.87943	0.01473
REACTOME_E2F_MEDIATED_REGULATION_OF_DNA_REPLICATION	-1.87878	0.014736
V$E2F_Q6_01	-1.87675	0.014958
WU_APOPTOSIS_BY_CDKN1A_VIA_TP53	-1.86938	0.016366
V$E2F_Q4	-1.86613	0.017122
GSE36476_CTRL_VS_TSST_ACT_72H_MEMORY_CD4_TCELL_YOUNG_DN	-1.86473	0.017343
V$E2F_Q6	-1.86231	0.017795
JACKSON_DNMT1_TARGETS_UP	-1.86236	0.017958
V$E2F1_Q3	-1.86101	0.018107
OXFORD_RALA_OR_RALB_TARGETS_UP	-1.85882	0.018468
GAL_LEUKEMIC_STEM_CELL_DN	-1.85771	0.01855
REACTOME_OLFACTORY_SIGNALING_PATHWAY	-1.85671	0.018677
CELL_CYCLE_PHASE	-1.85472	0.019103
GARGALOVIC_RESPONSE_TO_OXIDIZED_PHOSPHOLIPIDS_GREEN_UP	-1.85159	0.019629
MARSON_BOUND_BY_E2F4_UNSTIMULATED	-1.84778	0.020681
PUJANA_BRCA2_PCC_NETWORK	-1.84141	0.022741
MIKKELSEN_PLURIPOTENT_STATE_UP	-1.83779	0.023408
GSE3982_MAC_VS_TH1_DN	-1.83602	0.023412
REN_BOUND_BY_E2F	-1.83629	0.023444
WEST_ADRENOCORTICAL_TUMOR_MARKERS_UP	-1.83803	0.023501
BERENJENO_TRANSFORMED_BY_RHOA_UP	-1.83856	0.023603
ONDER_CDH1_TARGETS_1_DN	-1.83643	0.023619
SGCGSSAAA_V$E2F1DP2_01	-1.83459	0.023783
SHEPARD_BMYB_TARGETS	-1.83406	0.023806
TURASHVILI_BREAST_DUCTAL_CARCINOMA_VS_LOBULAR_NORMAL_UP	-1.83344	0.023828
GSE24634_TREG_VS_TCONV_POST_DAY7_IL4_CONVERSION_UP	-1.8322	0.024116
SENGUPTA_NASOPHARYNGEAL_CARCINOMA_UP	-1.83052	0.024494
FRASOR_RESPONSE_TO_SERM_OR_FULVESTRANT_DN	-1.82939	0.024668
DAUER_STAT3_TARGETS_DN	-1.82871	0.024769
NADERI_BREAST_CANCER_PROGNOSIS_UP	-1.82703	0.025243
V$E2F1_Q6	-1.82417	0.025902
CHROMOSOMAL_PART	-1.82366	0.025946
PUJANA_BREAST_CANCER_WITH_BRCA1_MUTATED_UP	-1.82271	0.026028
BURTON_ADIPOGENESIS_PEAK_AT_24HR	-1.82124	0.026395
LU_TUMOR_VASCULATURE_UP	-1.81981	0.026758
ZHAN_MULTIPLE_MYELOMA_CD2_DN	-1.81788	0.027279
V$E2F1DP1RB_01	-1.81724	0.027341
GSE29614_CTRL_VS_DAY7_TIV_FLU_VACCINE_PBMC_DN	-1.8156	0.027703
MORI_IMMATURE_B_LYMPHOCYTE_DN	-1.80745	0.030029
NEGATIVE_REGULATION_OF_CYTOKINE_BIOSYNTHETIC_PROCESS	-1.80788	0.030096
BIOCARTA_NKT_PATHWAY	-1.80845	0.030105
MCDOWELL_ACUTE_LUNG_INJURY_UP	-1.80845	0.030319
SISTER_CHROMATID_SEGREGATION	-1.806	0.030501
LOPEZ_MESOTELIOMA_SURVIVAL_TIME_UP	-1.80246	0.031804

NAME	*NES*	*FDR q-value*
GSE29614_DAY3_VS_DAY7_TIV_FLU_VACCINE_PBMC_DN	-1.80131	0.031832
GSE22886_UNSTIM_VS_IL2_STIM_NKCELL_DN	-1.80164	0.031902
TANG_SENESCENCE_TP53_TARGETS_DN	-1.79979	0.032238
LY_AGING_MIDDLE_DN	-1.79848	0.03228
V$E2F4DP1_01	-1.79876	0.032331
GAVIN_FOXP3_TARGETS_CLUSTER_P6	-1.7971	0.032743
RUIZ_TNC_TARGETS_DN	-1.79579	0.033173
NEGATIVE_REGULATION_OF_MAP_KINASE_ACTIVITY	-1.79467	0.033194
BARIS_THYROID_CANCER_DN	-1.79476	0.033356
GSE13485_DAY3_VS_DAY7_YF17D_VACCINE_PBMC_DN	-1.79296	0.033658
WEI_MYCN_TARGETS_WITH_E_BOX	-1.79224	0.033784
CELLULAR_DEFENSE_RESPONSE	-1.79139	0.034031
CASORELLI_ACUTE_PROMYELOCYTIC_LEUKEMIA_DN	-1.78855	0.035011
CELL_CYCLE_CHECKPOINT_GO_0000075	-1.78712	0.03553
CHASSOT_SKIN_WOUND	-1.78383	0.036878
XU_HGF_SIGNALING_NOT_VIA_AKT1_48HR_DN	-1.78179	0.037432
SIMBULAN_UV_RESPONSE_NORMAL_DN	-1.7818	0.037657
BASAKI_YBX1_TARGETS_UP	-1.7801	0.037975
CERIBELLI_PROMOTERS_INACTIVE_AND_BOUND_BY_NFY	-1.77865	0.038379
GSE36476_CTRL_VS_TSST_ACT_40H_MEMORY_CD4_TCELL_YOUNG_DN	-1.77775	0.038655
GSE3982_EFF_MEMORY_CD4_TCELL_VS_TH1_DN	-1.77369	0.039578
PID_AURORA_B_PATHWAY	-1.77544	0.039641
BENPORATH_CYCLING_GENES	-1.77397	0.03966
ROVERSI_GLIOMA_LOH_REGIONS	-1.77476	0.039747
GSE10239_NAIVE_VS_KLRG1INT_EFF_CD8_TCELL_DN	-1.77415	0.039803
MITOTIC_CELL_CYCLE	-1.77165	0.040045
CHROMOSOMEPERICENTRIC_REGION	-1.77209	0.040051
KORKOLA_TERATOMA	-1.77079	0.040263
KEGG_OLFACTORY_TRANSDUCTION	-1.77005	0.04039
SONG_TARGETS_OF_IE86_CMV_PROTEIN	-1.76934	0.040568
GSE30962_ACUTE_VS_CHRONIC_LCMV_SECONDARY_INF_CD8_TCELL_DN	-1.76059	0.044475
GSE1460_INTRATHYMIC_T_PROGENITOR_VS_NAIVE_CD4_TCELL_ADULT_BLOOD_UP	-1.7614	0.044513
V$E2F_02	-1.7609	0.044558
WONG_ENDMETRIUM_CANCER_UP	-1.75917	0.044947
MITOTIC_SISTER_CHROMATID_SEGREGATION	-1.75507	0.04706
TURASHVILI_BREAST_DUCTAL_CARCINOMA_VS_DUCTAL_NORMAL_UP	-1.75334	0.047908
LU_TUMOR_ENDOTHELIAL_MARKERS_UP	-1.75275	0.048044
KUROZUMI_RESPONSE_TO_ONCOCYTIC_VIRUS_AND_CYCLIC_RGD	-1.75083	0.048362
GCNP_SHH_UP_LATE.V1_UP	-1.75104	0.048485
MALIK_REPRESSED_BY_ESTROGEN	-1.75139	0.048525
REACTOME_PROCESSING_OF_CAPPED_INTRON_CONTAINING_PRE_MRNA	-1.74938	0.048894
V$E2F1DP1_01	-1.74752	0.049639

Gene sets are ordered according to FDR *q*-value. NES (normalized enrichment score) is also reported.

Supplementary Table 6B: Gene sets enriched in U266 treated cells

NAME	*NES*	*FDR q-value*
GARGALOVIC_RESPONSE_TO_OXIDIZED_PHOSPHOLIPIDS_RED_UP	2.351516	0
REACTOME_AMINO_ACID_SYNTHESIS_AND_INTERCONVERSION_TRANSAMINATION	2.37104	0
KRIGE_RESPONSE_TO_TOSEDOSTAT_6HR_UP	2.459397	0
MTOR_UP.N4.V1_UP	2.516844	0
PENG_LEUCINE_DEPRIVATION_UP	2.638843	0
KRIGE_RESPONSE_TO_TOSEDOSTAT_24HR_UP	2.659443	0
BHATI_G2M_ARREST_BY_2METHOXYESTRADIOL_DN	2.666824	0
TIEN_INTESTINE_PROBIOTICS_24HR_DN	2.67889	0
PODAR_RESPONSE_TO_ADAPHOSTIN_UP	2.694749	0
PACHER_TARGETS_OF_IGF1_AND_IGF2_UP	2.754331	0
BLUM_RESPONSE_TO_SALIRASIB_UP	2.762486	0
ZHAN_MULTIPLE_MYELOMA_CD1_UP	2.7971	0
HELLER_SILENCED_BY_METHYLATION_DN	2.81202	0
ZHAN_MULTIPLE_MYELOMA_CD1_VS_CD2_UP	2.872037	0
KRIGE_AMINO_ACID_DEPRIVATION	2.896582	0
KEGG_GLYCINE_SERINE_AND_THREONINE_METABOLISM	2.273052	3.01E-04
AMUNDSON_RESPONSE_TO_ARSENITE	2.241046	4.81E-04
TERAMOTO_OPN_TARGETS_CLUSTER_7	2.241159	5.09E-04
ONDER_CDH1_TARGETS_1_UP	2.22951	5.58E-04
WANG_HCP_PROSTATE_CANCER	2.19506	0.0012
FORTSCHEGGER_PHF8_TARGETS_UP	2.175743	0.00169
ALK_DN.V1_UP	2.171733	0.001789
KASLER_HDAC7_TARGETS_2_DN	2.157696	0.002089
PRAMOONJAGO_SOX4_TARGETS_UP	2.144733	0.002684
KAN_RESPONSE_TO_ARSENIC_TRIOXIDE	2.126259	0.003574
BMI1_DN_MEL18_DN.V1_UP	2.108819	0.004551
REACTOME_AMINO_ACID_TRANSPORT_ACROSS_THE_PLASMA_MEMBRANE	2.089989	0.006055
NEUTRAL_AMINO_ACID_TRANSMEMBRANE_TRANSPORTER_ACTIVITY	2.079356	0.006766
SCIBETTA_KDM5B_TARGETS_UP	2.075699	0.006995
XU_HGF_SIGNALING_NOT_VIA_AKT1_48HR_UP	2.064639	0.007916
IGARASHI_ATF4_TARGETS_DN	2.061486	0.008095
CONCANNON_APOPTOSIS_BY_EPOXOMICIN_UP	2.047539	0.009946
CHO_NR4A1_TARGETS	2.041657	0.010372
PARK_OSTEOBLAST_DIFFERENTIATION_BY_PHENYLAMIL_UP	2.019906	0.013799
NOJIMA_SFRP2_TARGETS_UP	2.008785	0.015458
HORIUCHI_WTAP_TARGETS_UP	2.009873	0.015707
SMITH_TERT_TARGETS_UP	2.005849	0.015714
FERRARI_RESPONSE_TO_FENRETINIDE_UP	1.992631	0.018693
MAHADEVAN_RESPONSE_TO_MP470_DN	1.964147	0.027304
HANN_RESISTANCE_TO_BCL2_INHIBITOR_DN	1.954325	0.030298
HUANG_FOXA2_TARGETS_UP	1.947281	0.032153
BOYAULT_LIVER_CANCER_SUBCLASS_G123_DN	1.948409	0.032375
KEGG_SELENOAMINO_ACID_METABOLISM	1.941641	0.033749
WANG_RESPONSE_TO_GSK3_INHIBITOR_SB216763_UP	1.939409	0.033834
GSE24634_IL4_VS_CTRL_TREATED_NAIVE_CD4_TCELL_DAY7_DN	1.932102	0.036692
HELLER_HDAC_TARGETS_SILENCED_BY_METHYLATION_DN	1.92893	0.037313
SASSON_FSH_RESPONSE	1.904675	0.04931

Gene sets are ordered according to FDR *q*-value. NES (normalized enrichment score) is also reported.

Supplementary Table 7: Clinical details of the 167 patients analyzed by NGS

Sample	*Sex*	*Age*	*Disease*	*Stage*[Δ]	*Phase*[‡]	*PP*[†]	*t(4;14)*	*t(11;14)*	*t(14;16)*	*t(14;20)*	*del(13)**	*del(17p)**	*1q gain**	*1p loss**	*HD°*
MM-004	F	58	MM	IA	D	Gκ	-	-	+	-	-	na	na	na	na
MM-015	M	71	MM	IIA	D	Gκ	-	+	-	-	-	-	-	-	-
MM-016	M	66	MM	IIIB	D	Gκ	-	-	-	-	-	-	+	-	+
MM-026	F	72	MM	IIIB	D	κ	-	+	-	-	-	-	-	na	na
MM-027	M	60	MM	IA	D	Gκ	-	-	-	-	+	-	na	na	na
MM-030	M	69	MM	IIIA	D	Gλ	-	-	-	-	-	-	-	+	+
MM-031	M	58	MM	IIIA	D	Aκ	-	+	-	-	-	-	+	+	-
MM-034	M	71	MM	IA	D	Gκ	-	-	-	-	-	-	-	-	+
MM-036	M	65	MM	IIA	D	Gκ	-	-	-	-	+	-	+	na	-
MM-037	F	50	MM	IIA	D	Gκ+Aκ	-	+	-	-	+	-	-	-	-
MM-038	F	67	MM	IIA	D	κ	-	-	-	-	+	-	-	-	+
MM-039	M	50	MM	IIA	D	Gλ	-	-	-	-	-	-	-	na	+
MM-042	M	54	MM	IIIA	D	Aλ	+	-	-	-	+	-	+	-	+
MM-043	F	73	MM	IA	D	Gκ+Gλ	-	-	-	-	-	-	+	-	-
MM-049	M	62	MM	IIIB	D	κ	-	-	-	-	-	-	-	-	+
MM-055	F	69	MM	IIIA	D	Gκ	-	+	-	-	-	-	-	na	-
MM-066	F	77	MM	IIIA	D	Aκ	+	-	-	-	-	-	-	na	-
MM-069	M	63	MM	IIA	D	Gκ	-	-	-	+	+	-	+	na	-
MM-078	F	59	MM	IIIA	D	κ	-	-	-	-	+	-	-	na	+
MM-079	F	74	MM	IIA	D	Gκ+Gλ	-	-	-	-	-	-	-	-	+
MM-087	F	84	MM	IIIA	D	Gλ	+	-	-	-	+	-	+	na	-
MM-115	F	53	MM	IIIA	D	Gλ	-	+	-	-	+	-	na	na	-
MM-123	M	55	MM	IIIA	D	Gκ	+	-	-	-	+	-	+	-	-
MM-131	M	73	MM	IA	D	Gκ	-	-	-	-	-	-	-	-	-
MM-140	M	62	MM	IIIB	D	Gκ	-	+	-	-	-	-	-	na	-
MM-143	M	61	MM	IIA	D	Gκ	-	-	-	-	-	-	+	-	+
MM-146	M	68	MM	IIA	D	Gλ	-	-	-	-	-	-	+	na	+
MM-148	F	55	MM	IA	D	Aκ	-	-	-	-	-	+	+	na	+
MM-149	F	52	MM	IA	D	Gλ	-	-	-	-	-	-	-	-	+
MM-150	F	68	MM	IIA	D	Gλ	-	-	-	-	-	-	+	-	+
MM-151	F	71	MM	IA	D	Gλ	-	-	-	-	-	-	-	-	+
MM-152	M	66	MM	IA	D	Gκ	-	-	-	-	-	-	-	na	+

Sample	*Sex*	*Age*	*Disease*	*Stage*[Δ]	*Phase*[‡]	*PP*[†]	*t(4;14)*	*t(11;14)*	*t(14;16)*	*t(14;20)*	*del(13)**	*del(17p)**	*1q gain**	*1p loss**	*HD°*
MM-154	F	71	MM	IIA	D	Gκ	-	-	+	-	+	-	+	-	-
MM-159	M	56	MM	IIA	D	κ	-	+	-	-	+	-	-	-	-
MM-174	M	85	MM	IIA	D	Aκ	-	-	-	-	-	-	-	-	+
MM-177	M	73	MM	IIIA	D	Gκ	-	-	-	-	+	-	+	-	-
MM-179	M	50	MM	IIIA	D	Gλ	-	+	-	-	-	+	-	-	+
MM-195	M	62	MM	IIA	D	Gκ	+	-	-	-	+	-	-	-	-
MM-200	F	63	MM	IA	D	Aλ	-	-	-	-	-	-	-	-	+
MM-202	M	64	MM	IIIA	D	Gκ	-	-	-	-	-	-	+	na	+
MM-206	F	73	MM	IIA	D	Gκ	+	-	-	-	+	-	-	+	-
MM-207	F	68	MM	IA	D	Aκ	-	-	-	-	+	-	+	-	-
MM-208	M	74	MM	IIA	D	Aλ	+	-	-	-	-	-	-	-	-
MM-209	F	65	MM	IIA	D	Gλ	-	-	-	-	-	-	-	-	+
MM-210	M	65	MM	IIB	D	Aλ	-	-	-	-	-	-	-	-	-
MM-212	F	55	MM	IIIA	D	Gκ	-	+	-	-	-	-	-	-	-
MM-213	M	66	MM	IIIA	D	λ	-	+	-	-	+	-	-	-	-
MM-219	M	73	MM	IIIA	D	Aλ	-	-	-	-	+	-	-	-	+
MM-224	F	52	MM	IIIA	D	Gκ	-	-	+	-	-	-	+	-	-
MM-229	M	75	MM	IIA	D	Gκ	-	-	-	-	-	-	-	-	-
MM-238	M	58	MM	IIB	D	Gκ	-	-	-	-	+	-	+	-	-
MM-239	F	72	MM	IIA	D	Aκ	-	-	-	-	+	-	-	-	+
MM-240	M	70	MM	IA	D	Gλ	-	-	-	-	-	-	-	-	+
MM-241	M	54	MM	IIA	D	Gκ	-	-	-	-	-	-	-	-	+
MM-242	M	69	MM	IIIA	D	Aκ	-	-	-	-	+	-	-	-	+
MM-243	F	68	MM	IIA	D	Gκ	-	-	-	-	-	-	+	-	+
MM-246	M	71	MM	IIIA	D	Aλ	-	+	-	-	-	-	-	-	-
MM-252	F	77	MM	IIA	D	Aλ	-	+	-	-	-	-	+	-	-
MM-253	F	45	MM	IIA	D	λ	-	-	-	-	+	-	+	-	+
MM-256	M	58	MM	IIIA	D	Aκ	-	-	+	-	+	-	-	-	-
MM-261	F	66	MM	IIB	D	Gκ	-	-	-	-	+	-	+	-	+
MM-262	M	74	MM	IA	D	λ	-	-	-	-	-	-	+	-	-
MM-263	F	65	MM	IIB	D	κ	+	-	-	-	+	-	+	-	-
MM-267	M	74	MM	IIIA	D	Gλ	-	-	-	-	+	-	-	-	+
MM-268	M	77	MM	IIIB	D	Gκ	-	-	-	-	-	-	-	-	-

Sample	*Sex*	*Age*	*Disease*	*Stage*$^{\Delta}$	*Phase*‡	*PP*†	*t(4;14)*	*t(11;14)*	*t(14;16)*	*t(14;20)*	*del(13)**	*del(17p)**	*1q gain**	*1p loss**	*HD°*
MM-269	F	67	MM	IIA	D	Gκ	-	-	-	-	+	-	-	-	+
MM-271	M	76	MM	IA	D	Gκ	-	-	-	-	+	-	-	-	-
MM-274	M	59	MM	IIA	D	Gκ	+	-	-	-	-	-	-	-	-
MM-276	F	70	MM	IIB	D	Gκ	+	-	-	-	+	-	-	-	-
MM-278	M	73	MM	IIA	D	Gλ	-	-	-	-	-	-	-	+	+
MM-279	F	71	MM	IIIA	D	Gλ	-	-	-	na	+	-	+	-	na
MM-280	M	62	MM	IIIA	D	Gλ	-	+	-	-	-	-	-	-	-
MM-281	F	77	MM	IIA	D	Gκ	-	-	-	-	-	-	-	-	-
MM-282	M	66	MM	IIIA	D	Gκ	-	-	-	-	-	-	+	-	+
MM-284	M	49	MM	IIIA	D	κ	-	+	-	-	na	-	-	-	-
MM-286	F	71	MM	IIA	D	Gκ	-	-	-	-	+	-	-	-	-
MM-295	F	74	MM	IIIA	D	λ	-	-	-	-	+	-	na	na	na
MM-300	M	65	MM	IIB	D	Gκ	-	-	-	-	-	-	na	na	na
MM-301	F	72	MM	IIA	D	Aλ	-	-	-	-	-	-	+	-	+
MM-302	M	65	MM	IA	D	Aκ	-	-	-	-	-	-	+	-	+
MM-308	M	58	MM	IA	D	Aκ	-	-	-	-	-	-	-	-	-
MM-313	M	66	MM	IIIA	D	Gλ	-	+	-	-	-	-	-	-	-
MM-310	M	67	MM	IIIA	D	Gλ	-	+	-	-	-	-	-	-	-
MM-314	F	70	MM	IIIA	D	Aλ	-	+	-	-	-	-	+	-	-
MM-317	M	56	MM	IIA	D	Gλ	-	+	-	-	-	-	+	-	-
MM-321	M	63	MM	IIIA	D	Gκ	-	-	-	-	+	-	-	-	+
MM-327	F	62	MM	IIA	D	absent	-	-	-	-	+	-	+	-	+
MM-330	F	61	MM	IIIA	D	Aκ	+	-	-	-	+	-	+	-	-
MM-334	F	45	MM	IA	D	Gλ	-	-	-	-	+	+	+	+	+
MM-335	F	68	MM	IIA	D	Aκ	-	-	+	-	+	-	-	-	-
MM-340	M	46	MM	IIIA	D	Gλ	-	+	-	-	+	-	-	-	-
MM-341	M	65	MM	IIIA	D	Gκ	-	-	-	-	-	-	+	-	-
MM-343	M	74	MM	IIIA	D	Aλ	-	+	-	-	-	-	-	-	-
MM-351	M	na	MM	IIA	D	Aκ	-	-	-	-	-	-	-	-	-
MM-362	F	80	MM	IIA	D	Gλ	-	-	-	-	+	+	+	-	-
MM-372	M	54	MM	IA	D	Gκ	-	+	-	-	-	-	+	-	-
MM-375	F	78	MM	IA	D	Gκ	+	-	-	-	-	+	-	-	-
MM-381	F	69	MM	IA	D	Gκ	-	-	-	na	-	-	-	-	na

Sample	Sex	Age	Disease	Stage[Δ]	Phase[‡]	PP[†]	t(4;14)	t(11;14)	t(14;16)	t(14;20)	del(13)*	del(17p)*	1q gain*	1p loss*	HD°
MM-382	M	85	MM	IIA	D	Gκ	-	-	-	-	+	+	na	na	na
MM-385	F	76	MM	IIIA	D	Gλ	-	-	-	-	-	-	-	-	+
MM-386	F	76	MM	IIA	D	Aλ	-	na	na	na	-	-	na	na	na
MM-387	F	44	MM	IIIA	D	Gκ	-	-	-	-	-	-	+	-	+
MM-392	F	53	MM	IA	D	Gλ	-	-	-	-	+	-	+	-	+
MM-398	F	65	MM	IIA	D	Gκ	-	+	-	-	+	-	+	-	-
MM-402	M	67	MM	IIA	D	Gκ	-	+	-	-	+	-	-	-	-
MM-405	M	69	MM	IA	D	Gκ	-	+	-	-	-	-	-	-	-
MM-406	M	62	MM	IIB	D	Aκ	-	+	-	-	-	-	+	-	na
MM-407	F	79	MM	IIIA	D	λ	-	-	-	-	+	-	-	-	-
MM-410	F	79	MM	IIIA	D	λ	-	-	-	-	-	-	+	-	-
MM-411	M	67	MM	IIIA	D	Gλ	-	-	-	-	-	-	-	-	+
MM-413	F	51	MM	IIA	D	Gκ	-	-	-	-	+	-	+	-	+
MM-414	F	67	MM	IIIA	D	Gλ	-	+	-	-	-	-	-	-	-
MM-422	F	74	MM	IIB	D	κ	-	-	-	-	+	-	-	-	-
MM-423	F	53	MM	IIA	D	λ	+	-	-	-	+	-	-	-	-
MM-424	F	84	MM	IA	D	Gκ	-	-	-	-	+	-	-	+	+
MM-425	F	65	MM	IIA	D	Aλ	-	-	-	-	+	-	+	-	-
MM-428	F	65	MM	IIA	D	Aλ	-	-	-	-	-	-	+	+	-
MM-429	F	63	MM	IIIA	D	Gκ	-	-	-	-	-	-	+	-	+
MM-430	na	62	MM	IIIA	D	Gλ	-	-	-	-	-	-	-	-	-
MM-431	F	70	MM	IA	D	Gλ	+	-	-	-	+	-	+	-	-
MM-433	M	72	MM	IIB	D	κ	-	+	-	-	-	-	-	-	-
MM-434	F	53	MM	IIA	D	κ	-	-	-	-	+	-	-	+	-
MM-435	na	42	MM	IIIA	D	Gλ	-	-	-	-	-	-	-	-	+
MM-437	F	72	MM	IIB	D	λ	-	-	-	-	+	-	+	-	-
MM-440	na	61	MM	IIA	D	Aκ	-	-	-	-	-	-	-	+	+
MM-441	na	na	MM	IA	D	Gλ	-	-	-	-	-	-	-	-	-
MM-442	F	65	sPCL	/	D	Gκ	-	-	-	-	+	-	+	+	-
MM-445	na	65	MM	IIA	D	κ	-	+	-	-	+	-	+	-	-
MM-446	na	52	MM	IIIA	D	Gλ	-	-	-	-	-	-	+	-	+
MM-447	na	57	MM	IIA	D	Gκ	-	+	-	-	-	-	+	-	-
MM-448	na	59	MM	IIA	D	Aκ	-	-	-	-	+	-	+	-	-

Sample	Sex	Age	Disease	Stage[Δ]	Phase[‡]	PP[†]	t(4;14)	t(11;14)	t(14;16)	t(14;20)	del(13)*	del(17p)*	1q gain*	1p loss*	HD°
MM-449	na	54	MM	IIA	D	Gκ	-	-	-	-	+	-	-	+	-
MM-464	M	75	MM	IIIA	D	Gλ	-	-	-	-	+	-	+	-	-
PCL-001	F	51	pPCL	/	D	κ	-	-	-	+	+	-	na	na	na
PCL-002	F	69	sPCL	/	R	λ	-	+	-	-	+	na	-	na	na
PCL-004	M	72	pPCL	/	D	Gκ	-	-	+	-	-	-	-	-	-
PCL-005	M	76	sPCL	/	R	Aκ	-	+	-	-	-	-	-	-	-
PCL-007	M	65	sPCL	/	R	κ	-	-	-	-	+	+	+	na	na
PCL-008	M	57	pPCL	/	D	Gλ	-	-	+	-	+	-	-	na	na
PCL-009	F	77	sPCL	/	R	Gκ	+	-	-	-	+	-	+	-	-
PCL-011	M	76	sPCL	/	R	Gκ	-	-	+	-	+	+	+	-	-
PCL-012	F	62	sPCL	/	R	Aκ	-	-	+	-	-	+	-	-	-
PCL-014	M	72	pPCL	/	D	λ	-	+	-	-	+	+	-	+	-
PCL-015	M	78	pPCL	/	D	κ	-	-	+	-	+	-	+	-	-
PCL-016	F	57	pPCL	/	D	Gκ	-	-	+	-	+	-	+	+	-
PCL-017	F	68	pPCL	/	D	Gκ	-	-	+	-	+	+	+	+	-
PCL-018	F	59	pPCL	/	D	κ	-	+	-	-	+	+	-	-	-
PCL-019	F	67	pPCL	/	D	Mκ	-	-	+	-	+	+	+	+	-
PCL-020	F	79	pPCL	/	D	Gλ	-	-	-	-	-	-	+	-	-
PCL-021	M	48	pPCL	/	D	Gλ	+	-	-	-	+	-	+	-	-
PCL-023	M	60	pPCL	/	D	Gκ	-	-	+	-	+	+	+	+	-
PCL-026	F	59	pPCL	/	D	Gκ	-	-	+	-	+	-	+	-	-
PCL-027	M	65	pPCL	/	D	λ	-	+	-	-	-	+	-	-	-
PCL-028	F	57	pPCL	/	D	κ	-	+	-	-	+	-	-	-	-
PCL-029	M	51	pPCL	/	D	Aλ	-	-	+	-	-	-	-	-	-
PCL-030	F	52	pPCL	/	D	κ	-	-	-	+	+	+	-	-	-
PCL-031	F	59	sPCL	/	D	Gλ	-	-	+	-	-	+	+	-	-
PCL-032	M	51	pPCL	/	D	Gκ	+	-	-	-	+	-	+	-	-
PCL-035	F	76	pPCL	/	D	κ	-	+	-	-	-	-	-	-	-
PCL-036	M	72	pPCL	/	D	Gκ	-	+	-	-	+	-	-	-	-
PCL-037	M	72	pPCL	/	D	Aλ	-	+	-	-	+	-	-	-	-
PCL-038	M	57	pPCL	/	D	Gκ	-	-	-	-	+	-	+	+	+
PCL-039	M	54	sPCL	/	D	Aκ	-	-	-	-	-	+	-	+	-
PCL-041	M	na	sPCL	/	D	Gκ	-	na	na	na	-	-	na	na	na

Sample	*Sex*	*Age*	*Disease*	*Stage*$^{\Delta}$	*Phase*‡	*PP*†	*t(4;14)*	*t(11;14)*	*t(14;16)*	*t(14;20)*	*del(13)**	*del(17p)**	*1q gain**	*1p loss**	*HD°*
PCL-042	F	69	sPCL	/	D	Gλ	-	+	-	-	-	-	-	-	-
PCL-043	F	68	pPCL	/	D	Gλ	-	+	-	-	-	-	-	-	-
PCL-046	F	50	pPCL	/	D	κ	-	-	-	-	+	-	+	-	+

$^{\Delta}$The Durie clinical staging system was adopted; ‡D: Diagnosis; R: relapse; †Paraprotein; *del(13), del(17), 1p loss and 1q gain were determined by FISH.
°HD = presence of the hyperdiploid status on the basis of FISH evaluation criteria.

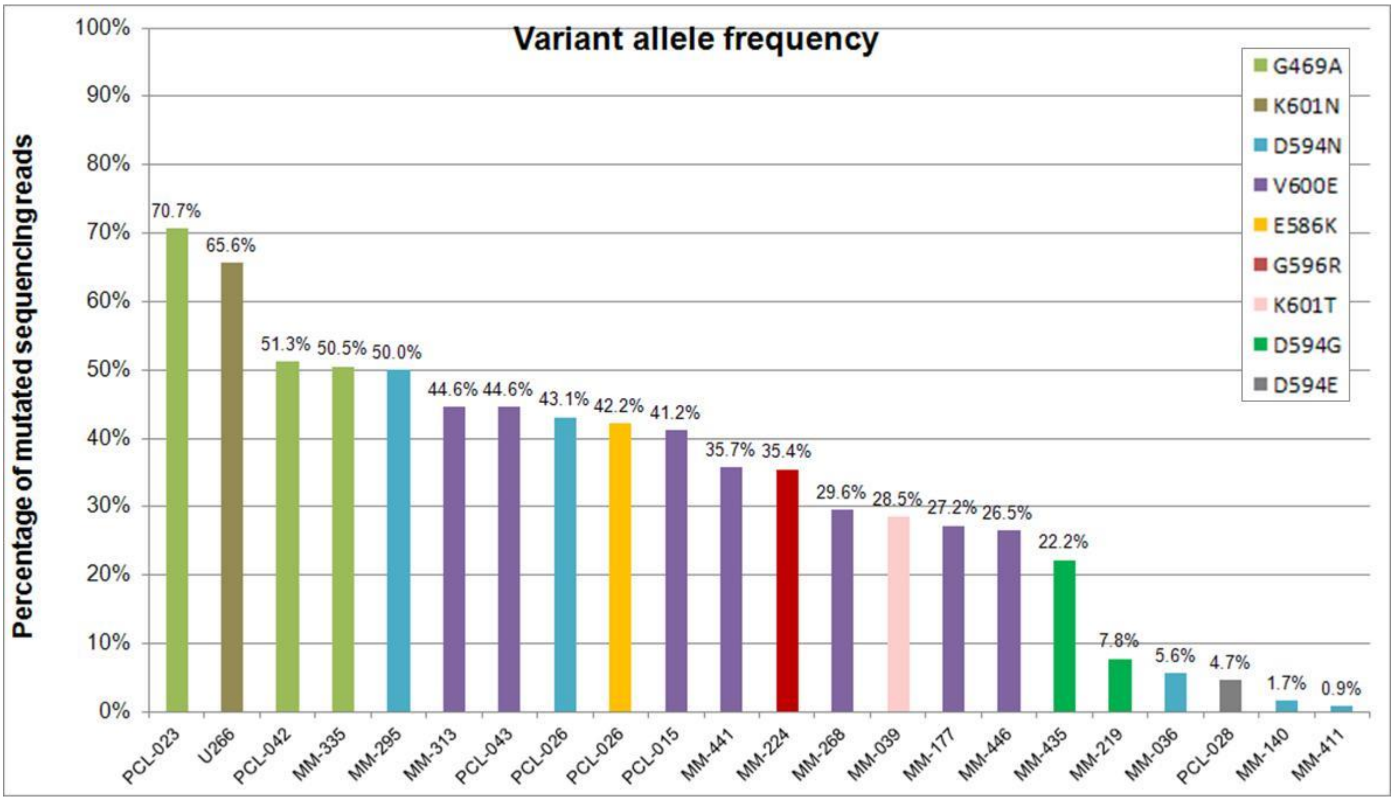

Supplementary Figure 1: Percentage of variant *BRAF* sequencing reads on total sequencing reads for the nine non-synonymous somatic mutations identified by NGS analysis. Horizontal axis: patients are ordered according to decreasing mutation load. Each color represents a different mutation (reported in the legend).

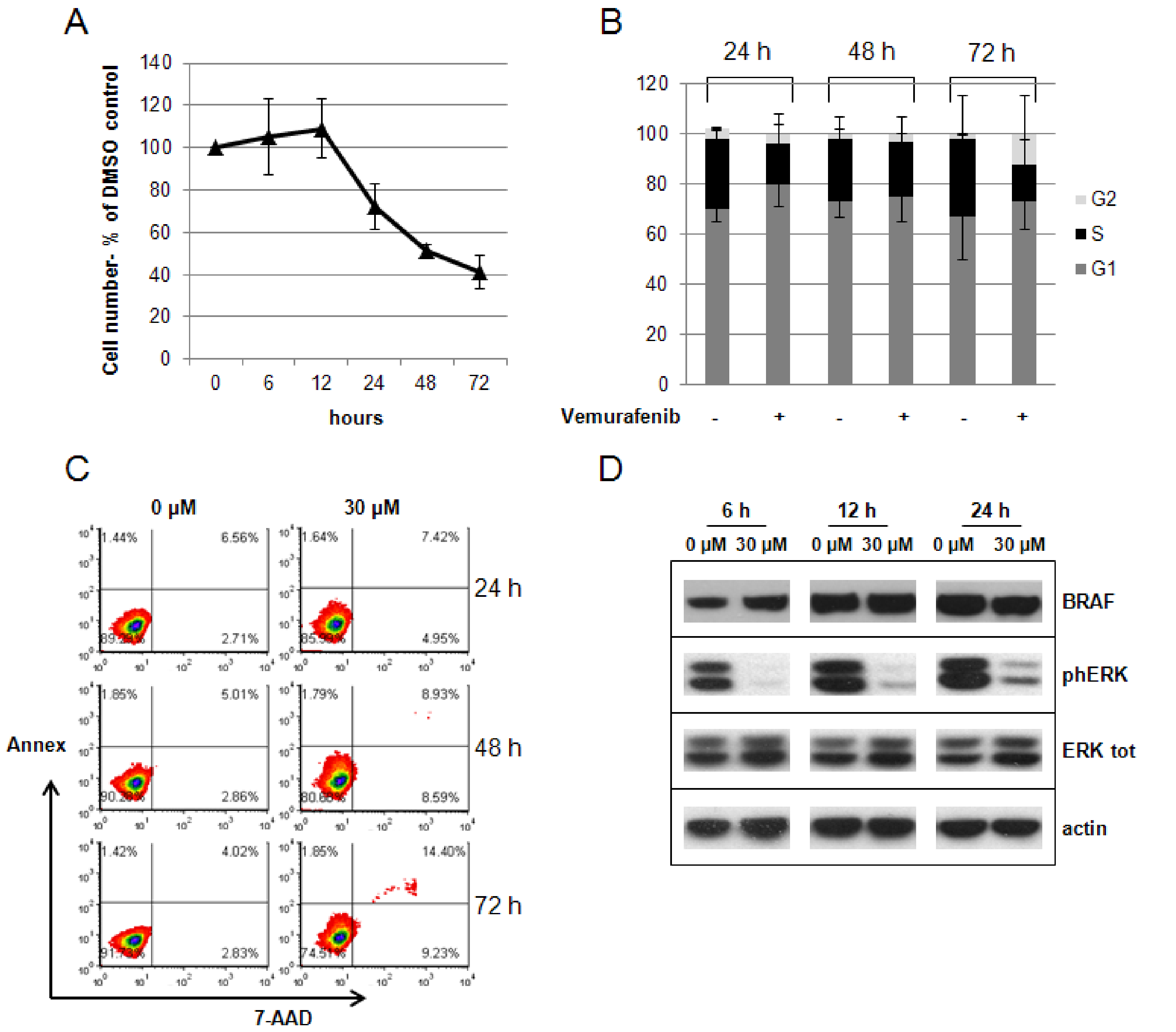

Supplementary Figure 2: (A) Effect of Vemurafenib on the growth of U266 cells assayed by the trypan blue dye exclusion test. Cells at a density of 0.5×10^6/ml were cultured for 72 h with Vemurafenib at the concentration of 30 µM. The data are presented as the mean±s.e. of three independent experiments.

(B) Bar graph representation of cell cycle of U266 cells treated with DMSO or with 30 µM Vemurafenib for 24, 48, and 72 hours, including standard deviations. Dark grey are cells in G1 phase, black are cells in S phase, light grey cells in G2 phase. The data are presented as the mean±s.e. of three independent experiments.

(C) Effect of Vemurafenib on the induction of apoptosis in U266 cells. Cells were cultured with Vemurafenib at the concentration of 30µM. Cells were stained with FITC-annexin V (AN) and 7-amino-actinomycin D (7AAD) at each time point, and analyzed by flow cytometry. Represented data are from one experiment representative of two independent experiments.

(D) Western blot analysis of U266 after 6-24 h of treatment with/without 30 µM Vemurafenib. Cell lysates were analyzed for the expression of BRAF, phospho-ERK1/2 (Thr 202/Tyr 204), and total ERK1/2.

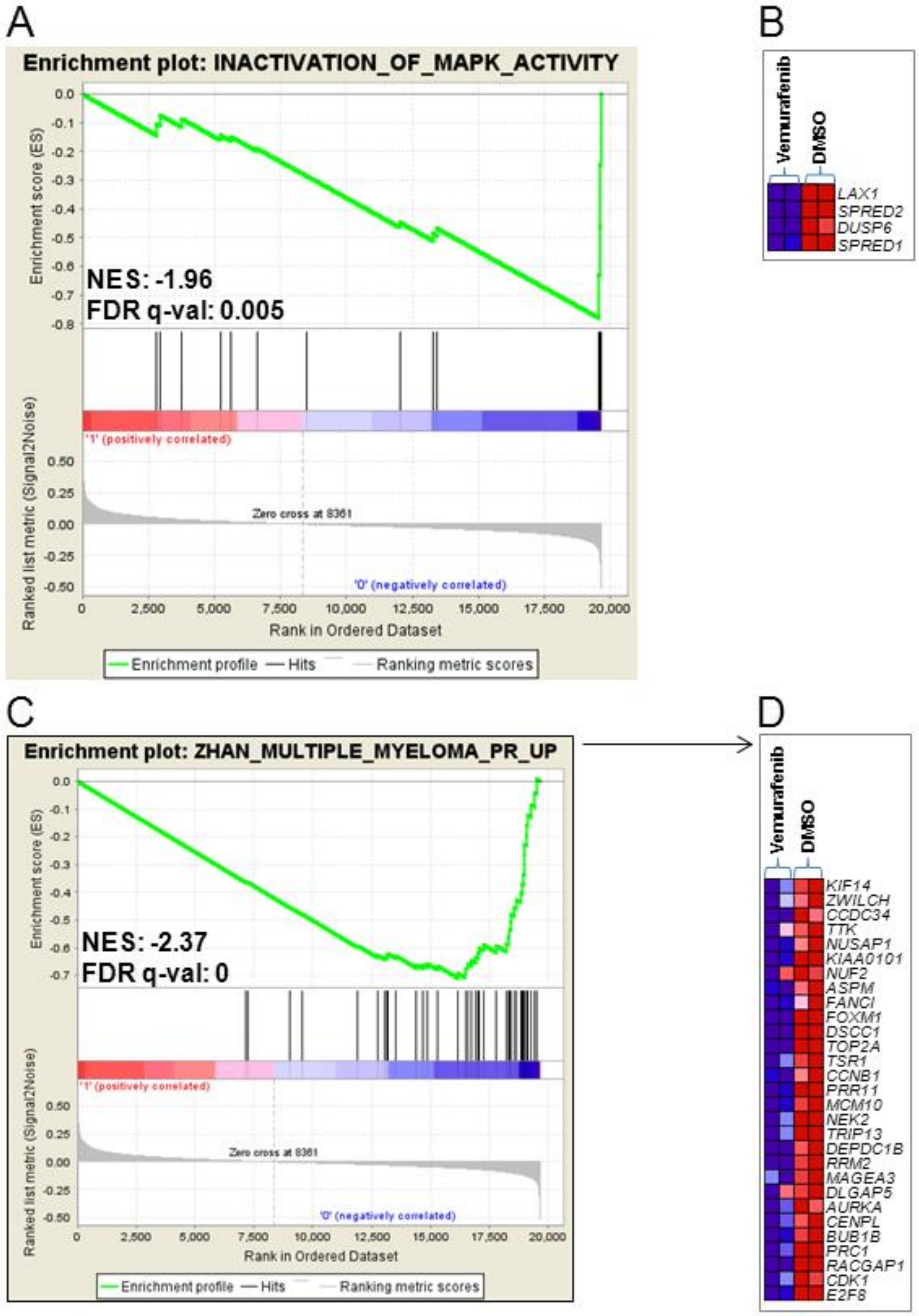

Supplementary Figure 3: (**A**, **C**) **GSEA enrichment plots of selected gene sets down-regulated in U266 cells treated with Vemurafenib with respect to DMSO controls.** The green curves show the enrichment score and reflect the degree to which each gene (black vertical lines) is represented at the bottom of the ranked gene list. Gene sets were considered enriched with an FDR <0.05. Normalized enrichment score (NES) and FDR are shown for both represented gene sets. **(B, D) Heat-maps in U266 treated and control cells of the genes constituting the leading edge subsets within the gene sets depicted in panels (A) and (C), respectively.**

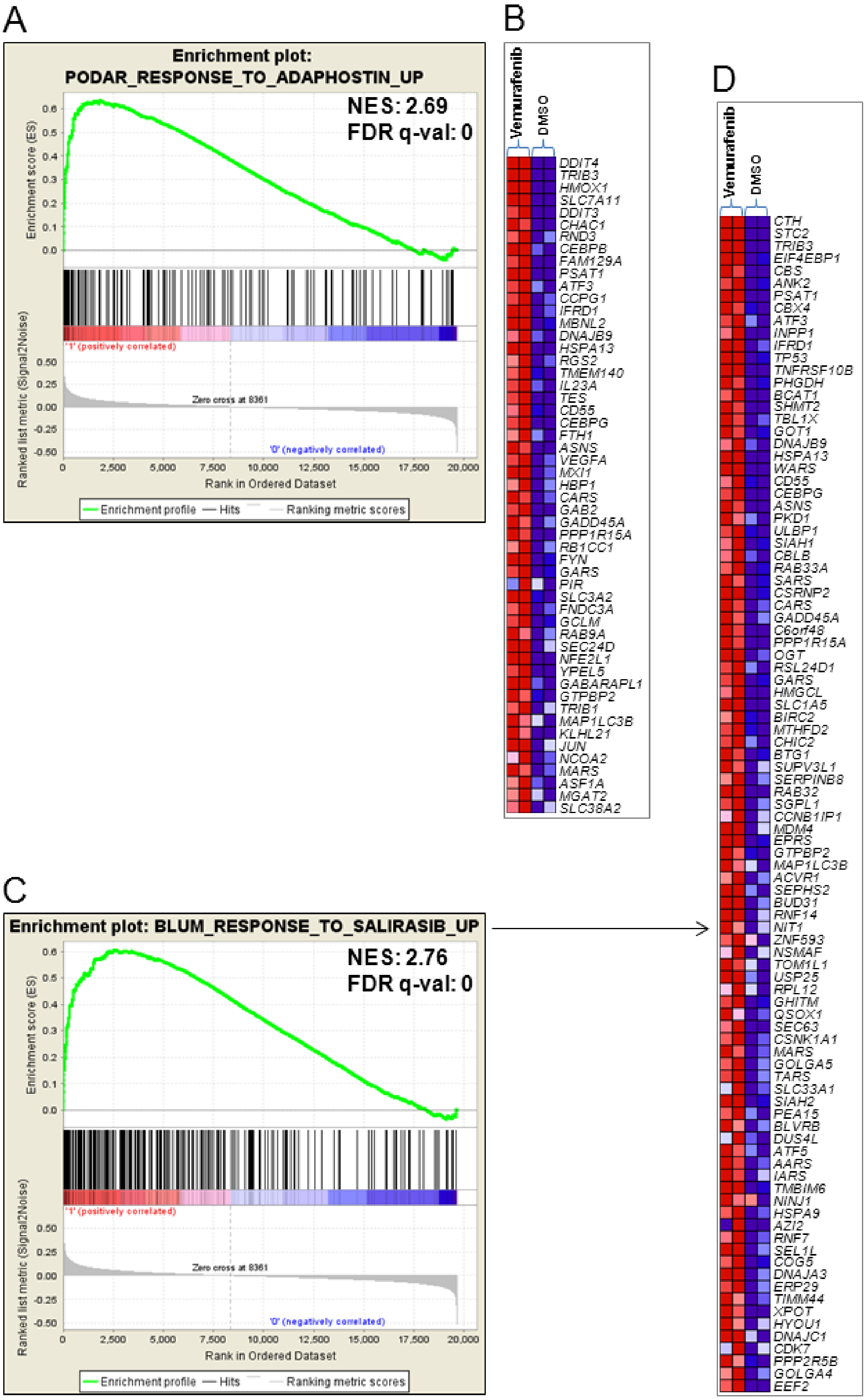

Supplementary Figure 4: (A, C) GSEA enrichment plots of selected gene sets up-regulated in U266 cells treated with Vemurafenib with respect to DMSO controls. The green curves show the enrichment score and reflect the degree to which each gene (black vertical lines) is represented at the top of the ranked gene list. Gene sets were considered enriched with an FDR <0.05. Normalized enrichment score (NES) and FDR are shown for both

represented gene sets. **(B, D) Heat-maps in U266 treated and control cells of the genes constituting the leading edge subsets within the gene sets depicted in panels (a) and (c), respectively.**
